# Bipolenins K–N: New sesquiterpenoids from the fungal plant pathogen *Bipolaris sorokiniana*

**DOI:** 10.3762/bjoc.15.198

**Published:** 2019-08-26

**Authors:** Chin-Soon Phan, Hang Li, Simon Kessler, Peter S Solomon, Andrew M Piggott, Yit-Heng Chooi

**Affiliations:** 1 School of Molecular Sciences, The University of Western Australia, Perth, WA 6009, Australia; 2 Research School of Biology, Australian National University, Canberra, ACT 2601, Australia; 3 Department of Molecular Sciences, Macquarie University, Sydney, NSW 2109, Australia

**Keywords:** *Bipolaris sorokiniana*, phytotoxicity, sesquiterpenes, terpenes

## Abstract

Chemical investigation of the barley and wheat fungal pathogen *Bipolaris sorokiniana* BRIP10943 yielded four new sativene-type sesquiterpenoid natural products, bipolenins K–N (**1**–**4**), together with seven related known analogues (**5**–**11**), and a sesterterpenoid (**12**). Their structures were determined by detailed analysis of spectroscopic data, supported by TDDFT calculations and comparison with previously reported analogues. These compounds were evaluated for their phytotoxic activity against wheat seedlings and wheat seed germination. The putative biosynthetic relationships between the isolated sesquiterpenoids were also explored.

## Introduction

Fungi belonging to the genus *Bipolaris* (teleomorph: *Cochliobolus*) have been reported to produce a diverse array of secondary metabolites, including sesquiterpenes [1–7], sesquiterpene-xanthones [8], diterpenes [9], sesterterpenes [10], cochlioquinones and peptides [11]. Moreover, several of these secondary metabolites are known to play important roles in mediating the virulence of these fungi against plant hosts [12]. Well-known examples include the host-specific toxins victorin and T-toxin and other non-host-specific toxins such as the ophiobolins [11]. *Bipolaris sorokiniana* (syn. *Cochliobolus sativus*) has been identified as the causative agent of multiple diseases on wheat and barley and is a major threat to yield improvement and food security in Central Asia [13]. Recent genome sequencing of 35 Australian strains of *B. sorokiniana* identified a known proteinaceous necrotrophic effector, *ToxA*, which confers host-specific virulence proteins and is proposed to be acquired through horizontal gene transfer [14]. To date, only three studies have explored phytotoxins from *B. sorokiniana* [2,7,10]. Therefore, in the framework of furthering our understanding of the roles of *B. sorokiniana* secondary metabolites in crop disease, we investigated the compounds produced by the *ToxA*-containing strain BRIP10943 (CS10) [14] and their phytotoxicity. This led to the isolation of four new sativene-type sesquiterpenoid natural products along with seven related known analogues and one sesterterpenoid. Herein, the isolation, structure elucidation and phytotoxic activities of these compounds are presented.

## Results and Discussion

**Figure 1:**
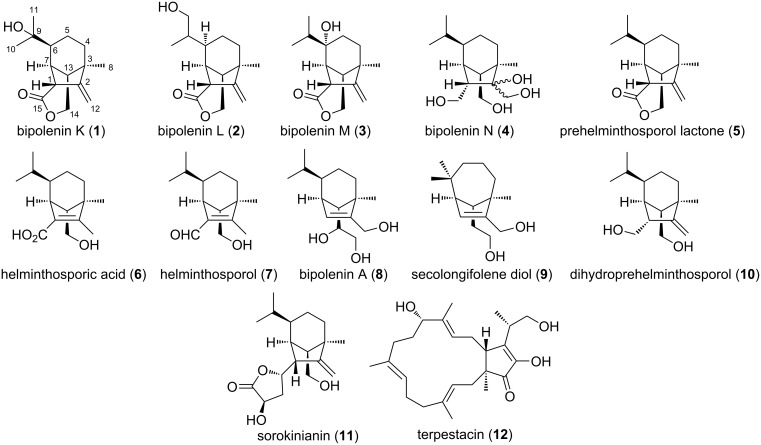
Structures of compounds **1**–**12** isolated from *B. sorokiniana*.

*B. sorokiniana* was cultivated for 22 days in Fries medium supplemented with rolled oats. The resulting broth and mycelia were extracted with methanol and the extracts were partitioned against EtOAc/MeOH/acetic acid (89.9:10:0.1 ratio). The combined organic layer was chromatographed repeatedly with silica gel and RP-HPLC to afford four new sativene-type sesquiterpenoids, bipolenins K–N (**1**–**4**), along with eight previously reported compounds (**5**–**12**), which were identified as sativene-type sesquiterpenoids prehelminthosporol lactone (**5**) [1], helminthosporic acid (**6**) [1], helminthosporol (**7**) [15], bipolenin A (**8**) [3], secolongifolene diol (**9**) [15], dihydroprehelminthosporol (**10**) [1] and sorokinianin (**11**) [2], and the cytotoxic sesterterpenoid, terpestacin (**12**) [16–17] (Figure 1).

**Figure 2:**
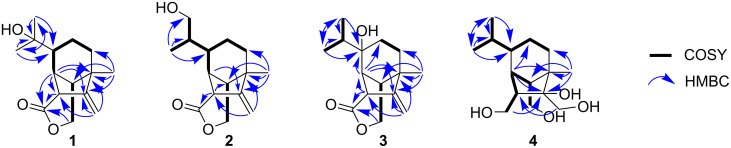
Key 2D NMR correlations of bipolenins K–N (**1**–**4**).

Bipolenin K (**1**) was isolated as a colourless oil. Its molecular formula was determined as C_15_H_22_O_3_ from the HRESIMS [M + H]^+^ ion at *m/z* 251.1646 (calcd for C_15_H_23_O_3_^+^, 251.1642), corresponding to five degrees of unsaturation. The IR absorption bands at 3445 and 1729 cm^−1^ revealed the presence of hydroxy and ester moieties, respectively. The ^13^C NMR spectrum (Figure S2, Supporting Information File 1) showed 15 distinct carbon signals, while ^13^C and ^1^H NMR data (Table 1) indicated an isopropyl unit (δ_C_ 28.3, 29.2 and 73.1; δ_H_ 1.20 and 1.24), one tertiary methyl (δ_C_ 20.1; δ_H_ 1.25), a disubstituted olefin (δ_C_ 105.7 and 155.2; δ_H_ 5.12 and 4.89) and four methines (δ_C_ 39.6, 51.1, 51.6 and 54.0; δ_H_ 1.73, 1.88, 2.65 and 3.77), which are typical resonances for sativene-type sesquiterpenoids. The NMR data for **1** were very similar to those for prehelminthosporol lactone (**5**) except for the replacement of a methine group (δ_C_ 32.1; δ_H_ 1.42) at C-9 in **5** with a hydroxylated quaternary carbon (δ_C_ 73.1) in **1**. This suggested that **1** was the 9-hydroxy analogue of **5**, which was further confirmed by detailed analysis of key 2D NMR correlations (Figure 2). Compound **1** was previously reported in 1970 as a semi-synthetic analogue of 9-hydroxyprehelminthosporol [18], but has not been previously isolated and characterised from a natural source.

**Table 1:** ^1^H and ^13^C NMR data for bipolenins K–N (**1**–**4**).

No.	Bipolenin K (**1**)^a^		Bipolenin L (**2**)^b^		Bipolenin M (**3**)^b^		Bipolenin N (**4**)^c^	

	δ_C_	δ_H_, mult (*J* in Hz)	δ_C_	δ_H_, mult (*J* in Hz)	δ_C_	δ_H_, mult (*J* in Hz)	δ_C_	δ_H_, mult (*J* in Hz)

1	54.0	3.77, br s	52.4	3.36, br s	53.1	3.31, m	47.4	2.19, dt (10.4, 3.1)
2	155.2		155.1		154.3		88.1	
3	46.3		46.0		45.6		50.0	
4	42.4	1.64, m	42.4	1.64, dd (12.8, 4.7)	39.3	1.85, td (14.8, 5.2)	30.6	1.60, m
		1.53, m		1.48, ddd (12.8, 5.6, 2.0)		1.34, m		
5	22.3	1.72, m	26.3	1.80, m	32.5	1.69, m	24.6	1.68, m
		1.52, m		1.27, m		1.34, m		1.31, m
6	51.1	1.73, m	41.9	1.70, m	74.8		45.8	1.18, m
7	39.6	2.65, br s	40.7	2.47, br s	46.3	2.44, br s	43.5	1.53, br s
8	20.1	1.25, s	20.3	1.25, s	20.2	1.25, s	19.4	0.98, s
9	73.1		39.7	1.44, m	35.8	1.67, septet (6.9)	30.4	1.34, m
10	29.2	1.24, s	15.8	1.02, d (6.9)	16.5	0.91, d (6.9)	21.4	0.91, d (6.9)
11	28.3	1.20, s	65.8	3.58, dd (10.8, 3.8)	16.5	0.91, d (6.9)	20.3	0.84, d (6.9)
				3.44, dd (10.8, 6.0)				
12	105.7	5.12, s	106.3	5.15, s	107.4	5.19, s	62.2	3.79, d (11.8)
		4.89, s		4.90, s		4.96, s		3.65, d (11.8)
13	51.6	1.88, dd (4.5, 1.8)	51.0	1.92, d (4.5)	46.0	2.38, d (4.4)	52.2	1.50, d (3.5)
14	71.3	4.46, d (11.7)	71.6	4.48, d (11.7)	72.1	4.53, d (11.7)	69.8	3.90, dd (7.8, 3.5)
		4.30, dd (11.7, 4.5)		4.30, dd (11.7, 4.5)		4.23, dd (11.7, 4.4)		3.36, d (7.8)
15	174.1		174.4		174.0		62.9	3.77, t (10.4)
								3.47, dd (10.4, 3.1)

^a^Recorded at 500/125 MHz for ^1^H/^13^C in CD_3_OD; ^b^Recorded at 600/150 MHz for ^1^H/^13^C in CD_3_OD; ^c^Recorded at 600/150 MHz for ^1^H/^13^C in CDCl_3_.

**Figure 3:**
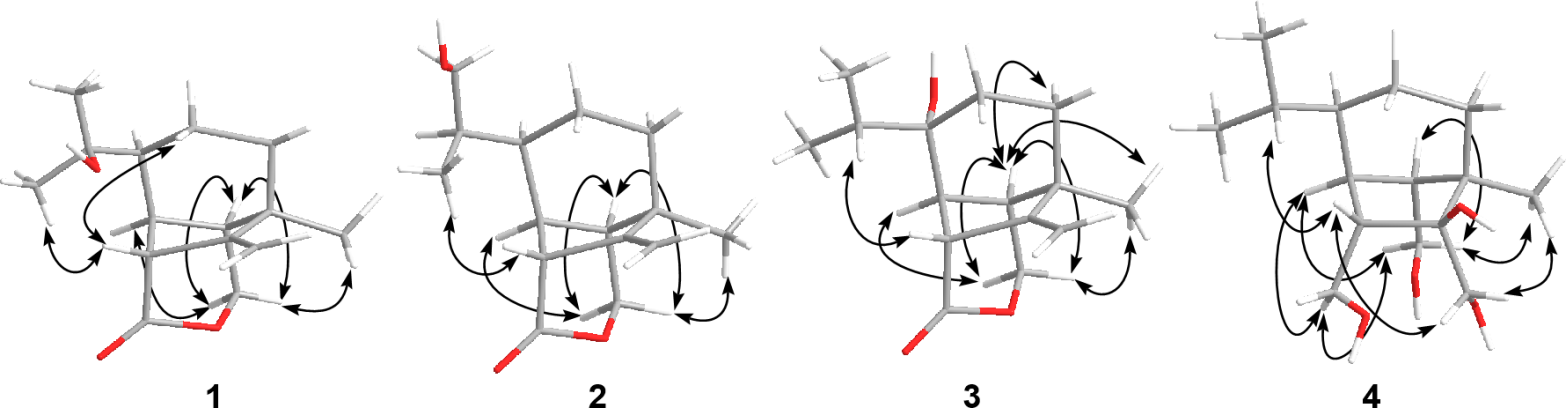
Key NOESY correlations of bipolenins K–N (**1**–**4**).

**Figure 4:**
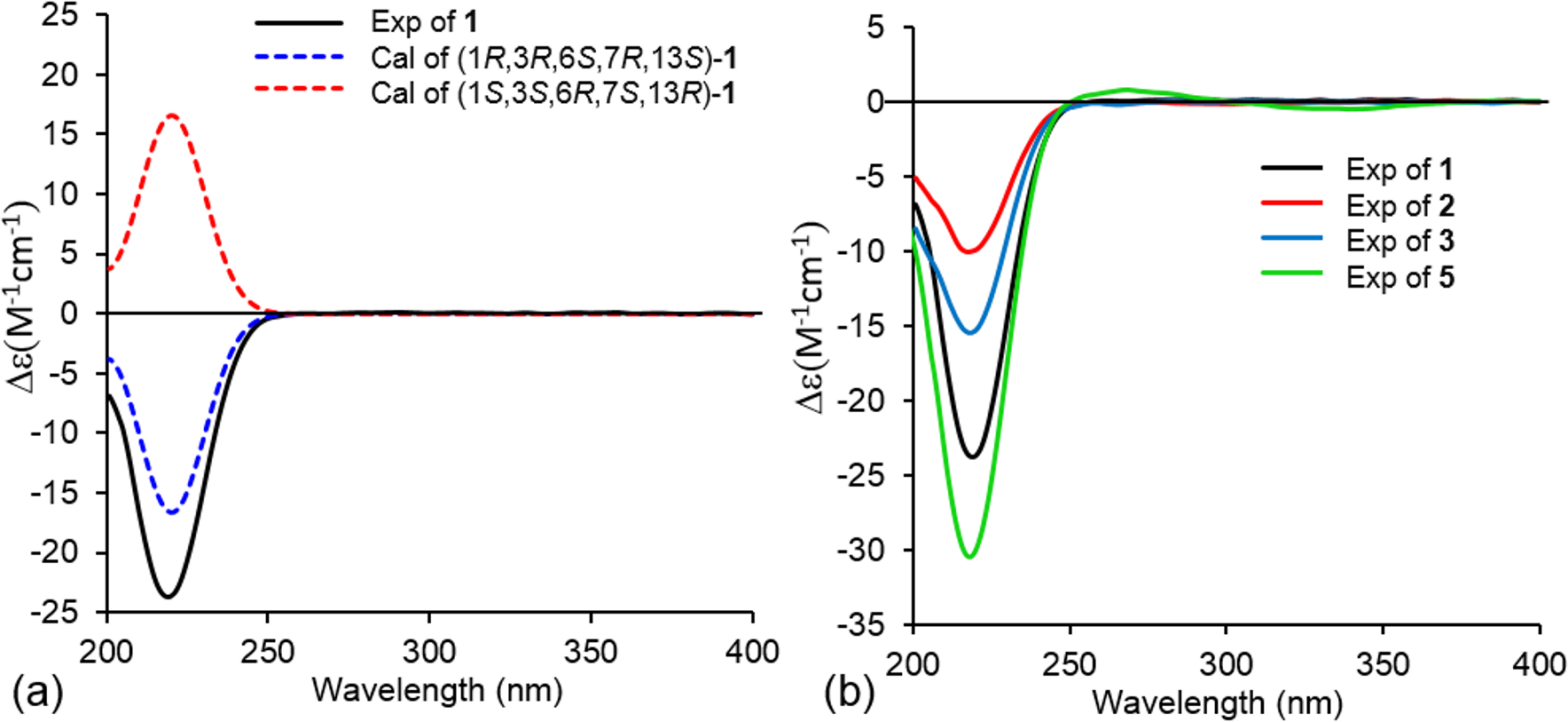
(a) Experimental ECD spectrum of **1** (MeOH) compared to TDDFT-calculated spectra (B3LYP-D3/def2-TZVPP) for the two possible enantiomers of **1**, which were blue-shifted by 9 nm. (b) Comparison of experimental ECD spectra of **1**–**3** and **5** (MeOH).

The relative configuration of **1** was established based on NOESY correlations (Figure 3) of H-1 to H_2_-5 and H_3_-10; and H_2_-14 to H-7, H_3_-8 and H-13. Due to the constrained bicyclo[3.2.1]octane ring system, these NOESY correlations indicated that H-1 was β-oriented, while H-6, H-7, H_3_-8 and H-13 were α-oriented. The absolute configuration of **1** was determined to be 1*R*,3*R*,6*S*,7*R*,13*S* by comparison of the experimental electronic circular dichroism (ECD) spectrum with time-dependent density functional theory (TDDFT)-calculated ECD spectra of the two possible enantiomers of **1** (Figure 4).

Bipolenin L (**2**) was isolated as a colourless oil. The HRESIMS [M + H]^+^ ion at *m*/*z* 251.1649 corresponded to a molecular formula C_15_H_22_O_3_ (calcd for C_15_H_23_O_3_^+^, 251.1642), which is isomeric with **1**. The ^1^H and ^13^C NMR data for **2** (Table 1) were also very similar to those for **5**, with the only significant difference being the presence of a hydroxymethylene group (δ_C_ 65.8; δ_H_ 3.58 and 3.44) in place of the methyl group at C-11. Thus, the structure of **2** was assigned as the 11-hydroxy analogue of **5**. The absolute configurations of the chiral centres in **2** were established to be the same as for **1** after investigation of the proton coupling constants (Table 1), NOESY correlations (Figure 3) and ECD spectra (Figure 4). The configuration at C-9 was not determined. Hence, structure **2** was determined as shown in Figure 1.

Bipolenin M (**3**) was purified as a colourless oil. The molecular formula C_15_H_22_O_3_ was based on a HRESIMS [M + H]^+^ ion at *m/z* 251.1647 (calcd for C_15_H_23_O_3_^+^, 251.1642), and is isomeric with **1** and **2**. The ^1^H and ^13^C NMR data for **3** (Table 1) were very similar to those for **5**, with the only significant difference being the replacement of the methine group (δ_C_ 47.1; δ_H_ 1.40) at C-6 in **5** with a hydroxylated quaternary carbon (δ_C_ 74.8) in **3**. This suggested that **3** was the 6-hydroxy analogue of **5**, which was further confirmed by detailed analysis of key 2D NMR correlations (Figure 2). The absolute configurations of the stereocentres in **3** were established to be identical to **1** and **2** based on the analysis of proton coupling constants (Table 1), NOESY correlations (Figure 3) and ECD spectra (Figure 4).

Bipolenin N (**4**) was acquired as a colourless oil. Its molecular formula was determined to be C_15_H_28_O_4_ from the HRESIMS [M + H − 2H_2_O]^+^ ion at *m/z* 237.1859 (calcd for C_15_H_25_O_2_^+^, 237.1849). The UV–vis spectra of **1**–**3** (Figure S37, Supporting Information File 1) and previously reported congener **5** were almost identical, while **4** displayed no significant UV–vis absorptions, suggesting the absence of both the ester and alkene moieties. This was confirmed by the analysis of the ^1^H and ^13^C NMR data for **4** (Table 1), which revealed the absence of ester and alkene resonances and the presence of three hydroxylated methylenes at C-12 (δ_C_ 62.2; δ_H_ 3.79 and 3.65), C-14 (δ_C_ 69.8; δ_H_ 3.90 and 3.36) and C-15 (δ_C_ 62.9; δ_H_ 3.77 and 3.47), and one hydroxylated quaternary carbon at C-2 (δ_C_ 88.1). This suggested **4** was related to **5**, but with reduction of the lactone ring to the dialcohol and dihydroxylation of the Δ^2,12^ double bond. Detailed analysis of the 2D NMR data for **4** (Figure 2) confirmed the seco-sativene-type scaffold. The relative configurations at C-1, C-3, C-6, C-7 and C-13 were determined to be the same as those of **1**–**3** and other reported analogues based on NOESY correlations (Figure 3), while the configuration at C-2 was not determined. The ECD spectrum of **4** (Figure S38, Supporting Information File 1) was measured, but no significant Cotton effect was observed. Therefore, the structure of **4** was determined as shown in Figure 1.

Equipped with the compounds, we tested **1**–**12** for phytotoxic activity against wheat seedlings. The compounds all showed negligible activities at 200 ppm, although **6** and **10** showed signs of necrosis at 500 ppm (Figure S40, Supporting Information File 1). In addition, the activities of **1**, **6**–**10** and **12** against wheat seed germination were also tested, with **7** inhibiting germination at 100 ppm (Figure S41, Supporting Information File 1). This corresponds to a previous report of the inhibitory effects of **7** on lettuce seed germination [19]. This activity could be due to the presence of an aldehyde moiety in **7**. Interestingly, an earlier study showed that **7** promoted the elongation of the shoots of rice seedlings [20]. Compound **12** was reported to have a broad spectrum of biological activities, including phytotoxicity on juvenile plant *Bromus tectorum* [21], syncytium formation inhibitory effects on cells infected with respiratory syncytial virus [22–23], induction of aerial mycelium formation in *Fusarium culmorum* [24], and as an inhibitor of ubiquinol-cytochrome c reductase binding protein, blocking mitochondrial ROS-mediated vascular endothelial growth factor receptor type 2 signalling pathways in endothelial cells [25]. However, **12** showed no activity against wheat seedlings or wheat seed germination in this study.

**Figure 5:**
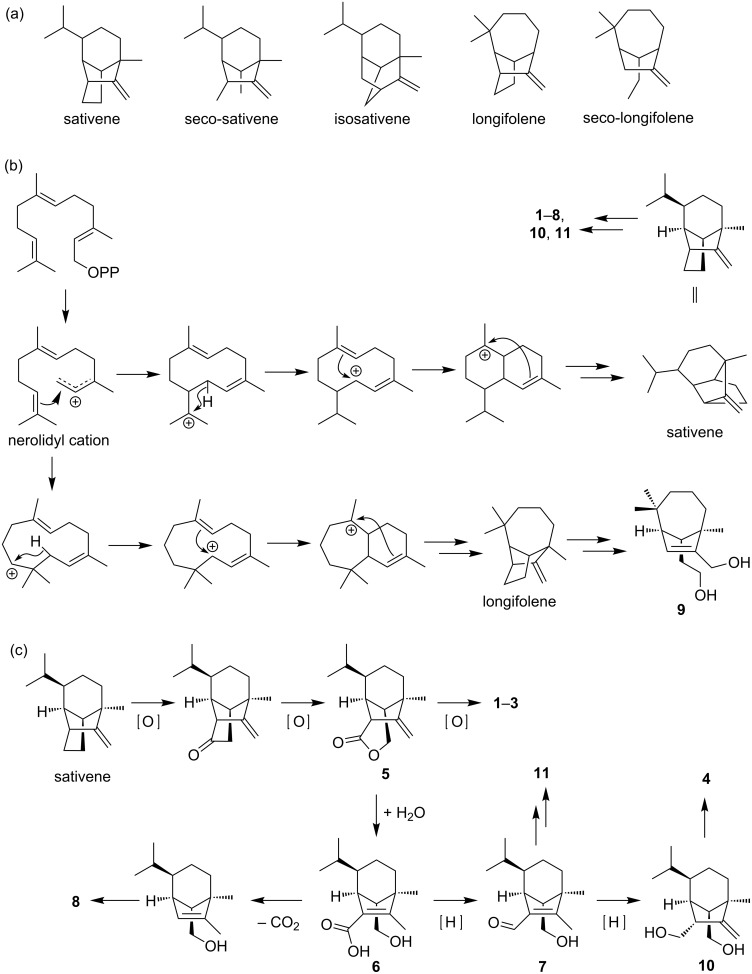
Relationship of sesquiterpenoids isolated in this study. A) Different groups of sativene/longifolene-type sesquiterpenoid scaffolds; B) The branched pathways to sativene- and longifolene-type sesquiterpenoids. C) Detailed proposed pathways to the sativene-derived sesquiterpenoids from this study.

The sativene-type sesquiterpenoids contain a bicyclo[3.2.1]octane backbone and are related to seco-sativene and isosativene scaffolds [15] (Figure 5a). They were also proposed to be related to the bicyclo[4.2.1]nonane-containing longifolene and seco-longifolene sesquiterpenoids, as they were often co-isolated [3–4,6,15,26–27]. A closer examination of the biosynthetic relationship between sativene and longifolene scaffolds suggests that the two pathways branched early at the nerolidyl cation (Figure 5b) [28–31]. The biosynthesis of **1**–**8** and **10**–**11** are likely to be derived from sativene with a key oxidation at C-15 followed by a Baeyer–Villiger oxidation to break the C-14–C-15 bond (Figure 5c). Based on an isotope labelling study, the γ-butyrolactone moiety on **11** has been proposed to be derived from oxaloacetic acid or similar TCA-cycle intermediates [32]. Compound **9**, which contains the seco-longifolene scaffold, is likely to be derived from longifolene via a similar Baeyer–Villiger mechanism proposed above for **1**–**8** and **10** and **11**.

Several sativene-type sesquiterpenoids have been previously reported from fungi, including from *Bipolaris* sp. [1], *B. sorokiniana* [2], *B. eleusines* [3–6,8], *Cochliobolus* sp. [26], *Cochliobolus sativus* [18], *Helminthosporium sativum* [20,33–34], *Drechslera* sp. [27], *Drechslera dematioidea* [15], and *Veronaea* sp. [35], most of which are Dothideomycetes. Significantly, this is the first report pertaining to sativene-type sesquiterpenoids from *B. sorokiniana* in 25 years, since the first and only literature account was published in 1994 [2]. Furthermore, structure **4** has a seco-sativene type scaffold without an olefin unit at C-1/C-2 or C-2/C-12. In contrast, all the previously known seco-sativene-type sesquiterpenoids possessed a double bond either at C-1/C-2 or C-2/C-12 [1–2,15,18,20,33], except drechslerine C, which contains a decarboxylated seco-sativene-type scaffold [15]. To the best of our knowledge, the previously reported sativene-type sesquiterpenoids **5** (isolated from *B. eleusines* and *Cochliobolus* sp., and from semi-synthetic analogue of prehelminthosporol with pyridinium chlorochromate or chromic acid) [1,4,18,26], **8** (isolated from *B. eleusines*) [3,6], and **10** (isolated from *Bipolaris* sp. and *Cochliobolus* sp.) [1,26], were reported in *B. sorokiniana* for the first time, while, known metabolites **6** and **7** [20], **9** [34], **11** [2], and **12** [16] were previously reported from *B. sorokiniana* (syn. *C. sativus* and *H. sativum*).

The terpene synthase responsible for the biosynthesis of the sativene/longifolene backbone of **1**–**11** remains unknown. Given that the genome of *B. sorokiniana* BRIP10943 has been sequenced [14], we surveyed the genome for potential terpene synthases that may be responsible for the biosynthesis of these compounds. Four putative sesquiterpene synthases were found, corresponding to the genes COCSADRAFT_31812, COCSADRAFT_346586, COCSADRAFT_83129 and COCSADRAFT_26102 annotated in the published genome *B. sorokiniana* ND90Pr in GenBank. However, it is difficult to determine which sesquiterpene synthase is responsible for biosynthesis of the sativene-type sesquiterpene backbone at this stage.

The biosynthetic gene cluster (*tpc*) for terpestacin (**12**) has been recently identified from *Bipolaris maydis* [36]. A didomain sesterterpene synthase (*tpcA*) with a terpene cyclase domain and polyprenyltransferase domain was demonstrated to be responsible for the production of the sesterterpene backbone of **12**. A BLASTp search using *tpcA* as query against the genome of *B. sorokiniana* ND90Pr and BRIP10943 identified COCSADRAFT_342920 in ND90Pr (and its homolog in BRIP10943), which shares 96% identity to *tpcA*. In the vicinity of the sesterterpene synthase gene, we also identified homologs for the two P450 oxygenases (*tpcB* and *tpcC*) encoded in the *tpc* cluster as COCSADRAFT_342924 (92% identity) and COCSADRAFT_146541 (98% identity), respectively, while the *tpcD* homolog in *B. sorokiniana* corresponded to COCSADRAFT_94398 (91% identity). This suggests that the homologous gene cluster in *B. sorokiniana* is likely responsible for production of **12**. We are currently investigating the genetic basis for the biosynthesis of the sativene-type terpenoid compounds identified from *B. sorokiniana*.

## Conclusion

Following the first and only reported isolation of a sativene-type sesquiterpenoid, sorokinianin (**11**), from *B. sorokiniana* in 1994 [2], we have expanded the number of reported analogues to eleven. These include the new sesquiterpenoid natural products, bipolenins K–N (**1**–**4**), as well as the previously reported sesquiterpenoids prehelminthosporol lactone (**5**), helminthosporic acid (**6**), helminthosporol (**7**), bipolenin A (**8**), secolongifolene diol (**9**), dihydroprehelminthosporol (**10**) and sorokinianin (**11**), together with a sesterterpenoid, terpestacin (**12**). We demonstrated that **6** and **10** have weak necrotic activity against wheat leaves, while **7** inhibited wheat seed germination at 100 ppm. These compounds served as markers for identifying putative sesquiterpene synthase genes in the genome of *B. sorokiniana* BRIP10943, allowing the molecular genetic basis for their biosynthesis and their roles in mediating the virulence of *B. sorokiniana* against wheat to be explored.

## Experimental

### General experimental procedures

Optical rotations were measured on an A. Krüss Optronic P8000 polarimeter. The IR spectra were collected on a Perkin Elmer Spectrum One FTIR spectrometer. The HR-ESIMS spectra were recorded on a Waters LCT Premier XE mass spectrometer. The ESIMS spectra were recorded on an Agilent 1260 LC system equipped with a DAD detector and coupled to an Agilent 6130 Quadrupole MS with an ESI source. The NMR spectra were recorded on Bruker Avance III HD 500 or AV600 spectrometers. The ECD spectra were recorded on a Jasco J-810 spectropolarimeter with MeOH as solvent. Flash cartridge (Reveleris, HP-silica, 12 g, 20 µm), Kinetex C18 (Phenomenex, 2.6 µm, 2.1 × 100 mm), and semi-preparative C18 (Grace, 5 µm, 10 × 250 mm) were used. All solvents used for extraction were analytical grade, and solvents for HPLC were HPLC grade.

### Biological material

The fungal strain *B. sorokiniana* BRIP10943 was obtained from Queensland Plant Pathology Herbarium (BRIP). It was isolated from a wheat field at Hermitage, QLD, Australia. The fungus was maintained on potato dextrose agar (PDA).

### Extraction and isolation

*B. sorokiniana* was cultured on 16 plates of V8PDA at 25 °C for 14 days, then inoculated in 4 L shake-flask culture (25 °C, 180 rpm for 22 days) in Fries medium supplemented with oat. The Fries medium was filtered and extracted by partition with EtOAc/MeOH/acetic acid at 89.9:10:0.1 ratio. The cells were extracted with MeOH and partition with EtOAc/MeOH/acetic acid at 89.7:10:0.3 ratio. Both organic partitioned layers were combined to obtain a light-yellow crude extract (205 mg), which was fractionated on a Reveleris flash chromatography (Grace) using gradient mode of H_2_O/MeOH equipped with the flash cartridge, UV and evaporative light scattering detector. The resulting fractions were further purified by RP-HPLC on gradient mode of H_2_O/MeCN equipped with the C_18_ column, and DAD detector to yield **1** (1.5 mg), **2** (0.4 mg), **3** (0.4 mg), **4** (0.4 mg), **5** (0.5 mg), **6** (1.0 mg), **7** (4.0 mg), **8** (1.8 mg), **9** (2.8 mg), **10** (3.0 mg), **11** (0.4 mg), and **12** (1.3 mg). The Kinetex C18 on RP-HPLC (Phenomenex, 2.6 µm, 2.1 × 100 mm, 0.75 mL/min, DAD detection 200–800 nm, gradient: 0–10 min 5–95% MeCN with 0.1% formic acid, 10–15 min 95% MeCN with 0.1% formic acid) eluted **1** (*t*_R_ 5.10 min), **2** (*t*_R_ 4.79 min), **3** (*t*_R_ 5.28 min), **4** (*t*_R_ 5.96 min), **5** (*t*_R_ 7.64 min), **6** (*t*_R_ 6.00 min), **7** (*t*_R_ 6.45 min), **8** (*t*_R_ 4.85 min), **9** (*t*_R_ 5.69 min), **10** (*t*_R_ 6.37 min), **11** (*t*_R_ 6.48 min) and **12** (*t*_R_ 6.88 min).

Bipolenin K (**1**): Colourless oil; [α]_D_^20^ −59 (*c* 0.15, MeOH); IR (KBr) λ_max_ 3445, 2926 and 1729 cm^−1^; ^1^H and ^13^C NMR data, see Table 1; HRESIMS *m*/*z*: [M + H]^+^ calcd for C_15_H_23_O_3_^+^, 251.1642; found, 251.1646, and [M + H − H_2_O]^+^ calcd for C_15_H_21_O_2_^+^, 233.1536; found, 233.1531.

Bipolenin L (**2**): Colourless oil; [α]_D_^20^ −63 (*c* 0.04, MeOH); IR (KBr) λ_max_ 3419, 2920 and 1730 cm^−1^; ^1^H and ^13^C NMR data, see Table 1; HRESIMS *m*/*z*: [M + H]^+^ calcd for C_15_H_23_O_3_^+^, 251.1642; found, 251.1649, and [M + H − H_2_O]^+^ calcd for C_15_H_21_O_2_^+^, 233.1536; found, 233.1535.

Bipolenin M (**3**): Colourless oil; [α]_D_^20^ −57 (*c* 0.04, MeOH); IR (KBr) λ_max_ 3418, 2927 and 1720 cm^−1^; ^1^H and ^13^C NMR data, see Table 1; HRESIMS *m*/*z*: [M + H]^+^ calcd for C_15_H_23_O_3_^+^, 251.1642; found, 251.1647, and [M + H − H_2_O]^+^ calcd for C_15_H_21_O_2_^+^, 233.1536; found, 233.1545.

Bipolenin N (**4**): Colourless oil; [α]_D_^20^ +38 (*c* 0.04, MeOH); IR (KBr) λ_max_ 3334, 2927 and 1045 cm^−1^; ^1^H and ^13^C NMR data, see Table 1; HRESIMS *m*/*z*: [M + H − 2H_2_O]^+^ calcd for C_15_H_25_O_2_^+^, 237.1849; found, 237.1859.

### Phytotoxicity assays

The phytotoxicity assays on wheat leaves and seeds were carried out as previously reported [37]. Briefly, the leaves of 17-days-old wheat seedlings in 10 cm planting pots were grown at 20 °C under a 16 h/8 h light/dark cycle regime. Compounds were dissolved in 0.2% MeOH/H_2_O and 30 µL of dissolved solution was infiltrated on the adaxial face of leaves at concentrations of 100, 200 and 500 ppm (serial dilution) using a 1 mL syringe. The leaves were examined for the presence of necrosis or chlorosis after 24 h and 48 h. The control consisted of 30 µL of 0.2% MeOH/H_2_O without dissolved compound. Two wheat seeds (sterilised by 10% EtOH) were placed on top of the agar (1.5% agar in 1 mL of tap water) containing 100 ppm of compound. The control was agar containing 30 µL of MeOH. The seeds were monitored for the progress of germination on day 5 and day 7.

### Calculation of ECD spectra

Structures were initially subjected to a LowModeMD conformational search using the Molecular Operating Environment 2019.0101 package. The lowest energy geometry for each molecule was further optimised by DFT at the B3LYP-D3/def2-TZVPP level of theory using Turbomole 7.1 [38] and ECD spectra were calculated in Turbomole using TDDFT (B3LYP-D3/def2-TZVPP).

## Supporting Information

File 1 NMR, IR and MS spectra of compounds **1**–**4**.
