# Supplementary material for: Bipolenins K–N: New sesquiterpenoids from the fungal plant pathogen Bipolaris sorokiniana
Source: Beilstein J Org Chem. 2019 Aug 26;15:2020–8. doi: 10.3762/bjoc.15.198 (PMC6720731; doi:10.3762/bjoc.15.198)
Supplement: File 1 — NMR, IR and MS spectra of compounds 1–4. [file Beilstein_J_Org_Chem-15-2020-s001.pdf]

BEILSTEIN JOURNAL OF ORGANIC CHEMISTRY

Supporting Information

for

Bipolenins K–N: New sesquiterpenoids from the fungal plant pathogen *Bipolaris sorokiniana*

Chin-Soon Phan, Hang Li, Simon Kessler, Peter S. Solomon, Andrew M. Piggott and Yit-Heng Chooi

Beilstein J. Org. Chem. **2019,** *15,* 2020–2028. doi:10.3762/bjoc.15.198

NMR, IR and MS spectra of compounds 1–4

License and Terms: This is a supporting information file under the terms of the Creative Commons Attribution License (http://creativecommons.org/licenses/by/4.0). Please note that the reuse, redistribution and reproduction in particular requires that the authors and source are credited.
The license is subject to the *Beilstein Journal of Organic Chemistry* terms and conditions: (https://www.beilstein-journals.org/bjoc)

Contents

Figure S1: ^{1}H NMR spectrum (500 MHz) of **1** in CD_3OD

Figure S2: ^{13}C NMR spectrum (125 MHz) of **1** in CD_3OD

Figure S3: HSQC spectrum (500 MHz) of **1** in CD_3OD

Figure S4: HMBC spectrum (500 MHz) of **1** in CD_3OD

Figure S5: ^{1}H-^{1}H COSY spectrum (500 MHz) of **1** in CD_3OD

Figure S6: NOESY spectrum (500 MHz) of **1** in CD_3OD

Figure S7: HRESIMS spectrum of **1**

Figure S8: ESIMS spectrum of **1**

Figure S9: IR spectrum of **1**

Figure S10: ^{1}H NMR spectrum (600 MHz) of **2** in CD_3OD

Figure S11: ^{13}C NMR spectrum (150 MHz) of **2** in CD_3OD

Figure S12: HSQC spectrum (600 MHz) of **2** in CD_3OD

Figure S13: HMBC spectrum (600 MHz) of **2** in CD_3OD

Figure S14: ^{1}H-^{1}H COSY spectrum (600 MHz) of **2** in CD_3OD

Figure S15: NOESY spectrum (600 MHz) of **2** in CD_3OD

Figure S16: HRESIMS spectrum of **2**

Figure S17: ESIMS spectrum of **2**

Figure S18: IR spectrum of **2**

Figure S19: ^{1}H NMR spectrum (600 MHz) of **3** in CD_3OD

Figure S20: ^{13}C NMR spectrum (150 MHz) of **3** in CD_3OD

Figure S21: HSQC spectrum (600 MHz) of **3** in CD_3OD

Figure S22: HMBC spectrum (600 MHz) of **3** in CD_3OD

Figure S23: ^{1}H-^{1}H COSY spectrum (600 MHz) of **3** in CD_3OD

Figure S24: NOESY spectrum (600 MHz) of **3** in CD_3OD

Figure S25: HRESIMS spectrum of **3**

Figure S26: ESIMS spectrum of **3**

Figure S27: IR spectrum of **3**

Figure S28: ^{1}H NMR spectrum (600 MHz) of **4** in $CDCl_3$

Figure S29: ^{13}C NMR spectrum (150 MHz) of **4** in $CDCl_3$

Figure S30: HSQC spectrum (600 MHz) of **4** in $CDCl_3$

Figure S31: HMBC spectrum (600 MHz) of **4** in $CDCl_3$

Figure S32: ^{1}H-^{1}H COSY spectrum (600 MHz) of **4** in $CDCl_3$

Figure S33: NOESY spectrum (600 MHz) of **4** in $CDCl_3$

Figure S34: HRESIMS spectrum of **4**

Figure S35: ESIMS spectrum of **4**

Figure S36: IR spectrum of **4**

Figure S37: UV-vis spectrum of **1**–**3** and **5**

Figure S38: ECD spectrum of **4**

Figure S39: Phytotoxic activity of **1**–**12** against wheat leaves at 200 ppm

Figure S40: Phytotoxic activity of **1**–**10** and **12** against wheat leaves at 500 ppm

Figure S41: Phytotoxic activity of **1**, **6**–**10** and **12** against wheat germination at 100 ppm on day 5

Figure S42: Phytotoxic activity of **1**, **6**–**10** and **12** against wheat germination at 100 ppm on day 7

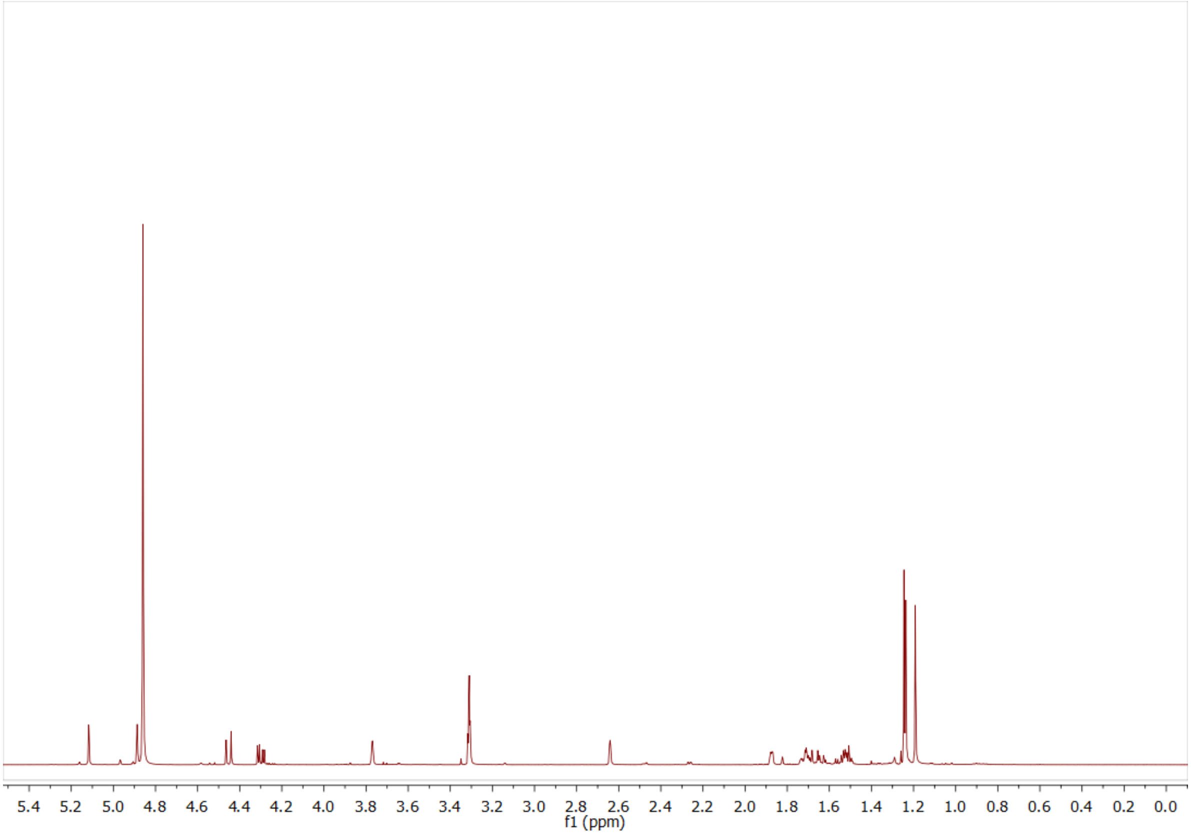

Figure S1: ^{1}H NMR spectrum (500 MHz) of **1** in CD_3OD

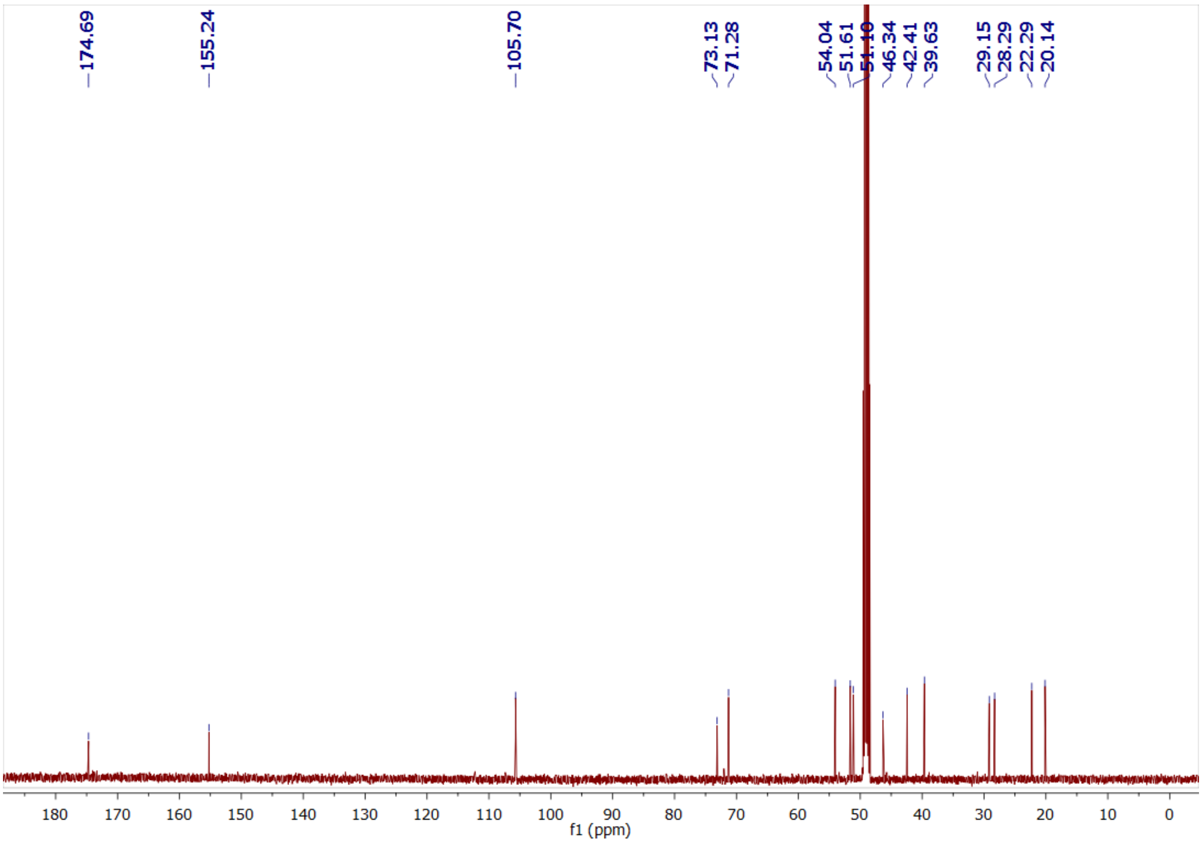

Figure S2: ^{13}C NMR spectrum (125 MHz) of **1** in CD_3OD

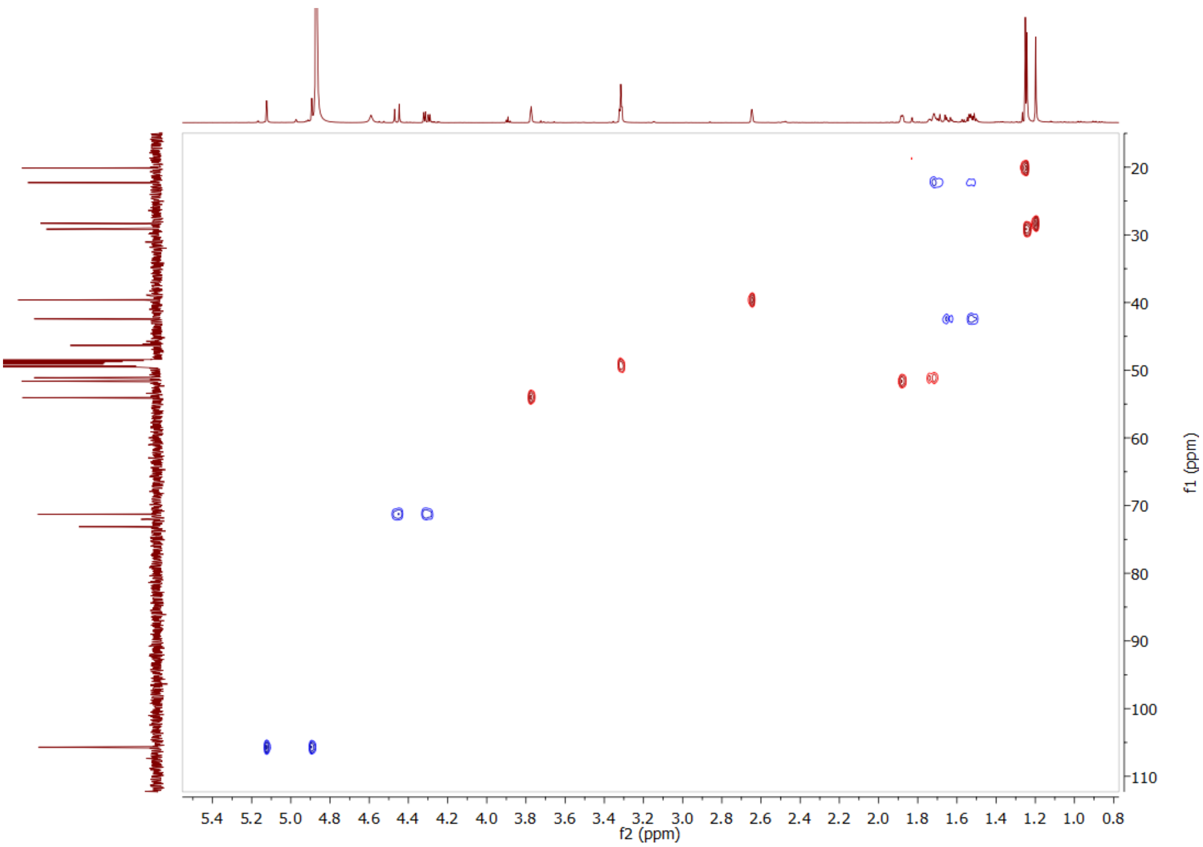

Figure S3: HSQC spectrum (500 MHz) of **1** in CD_3OD

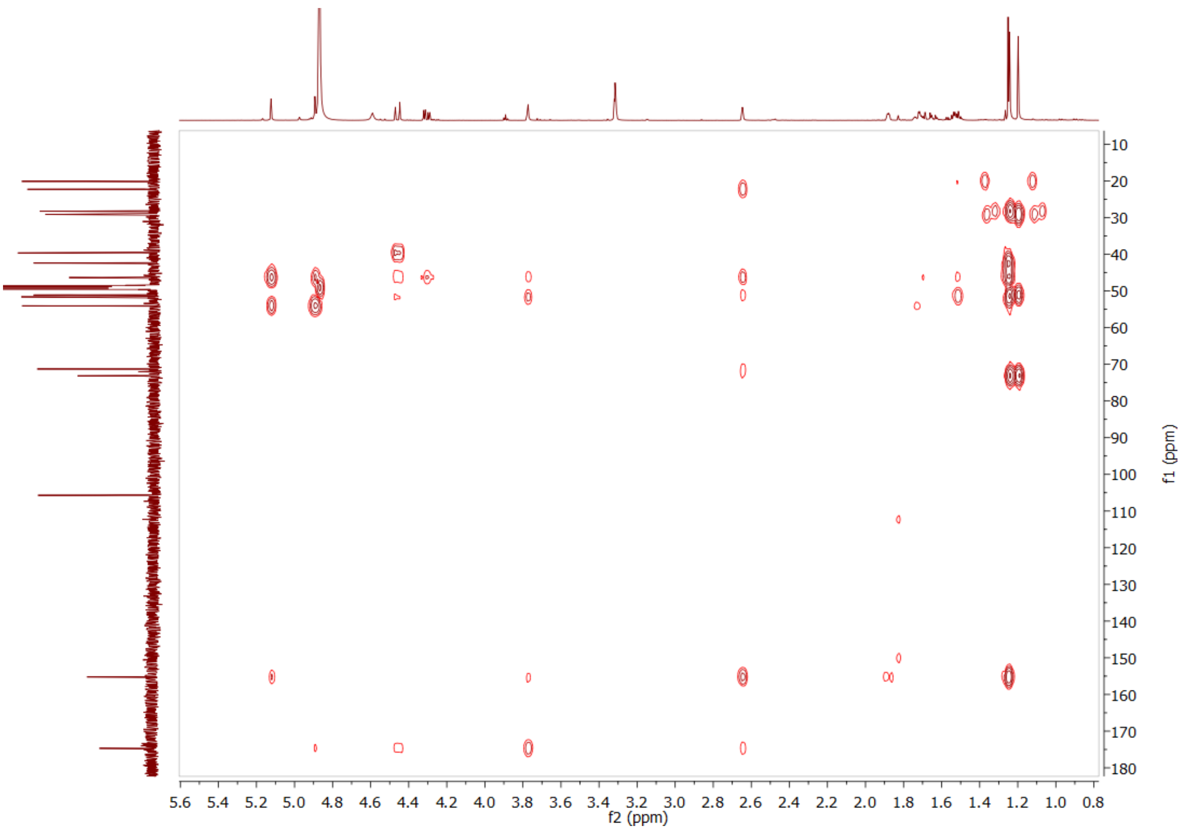

Figure S4: HMBC spectrum (500 MHz) of **1** in CD_3OD

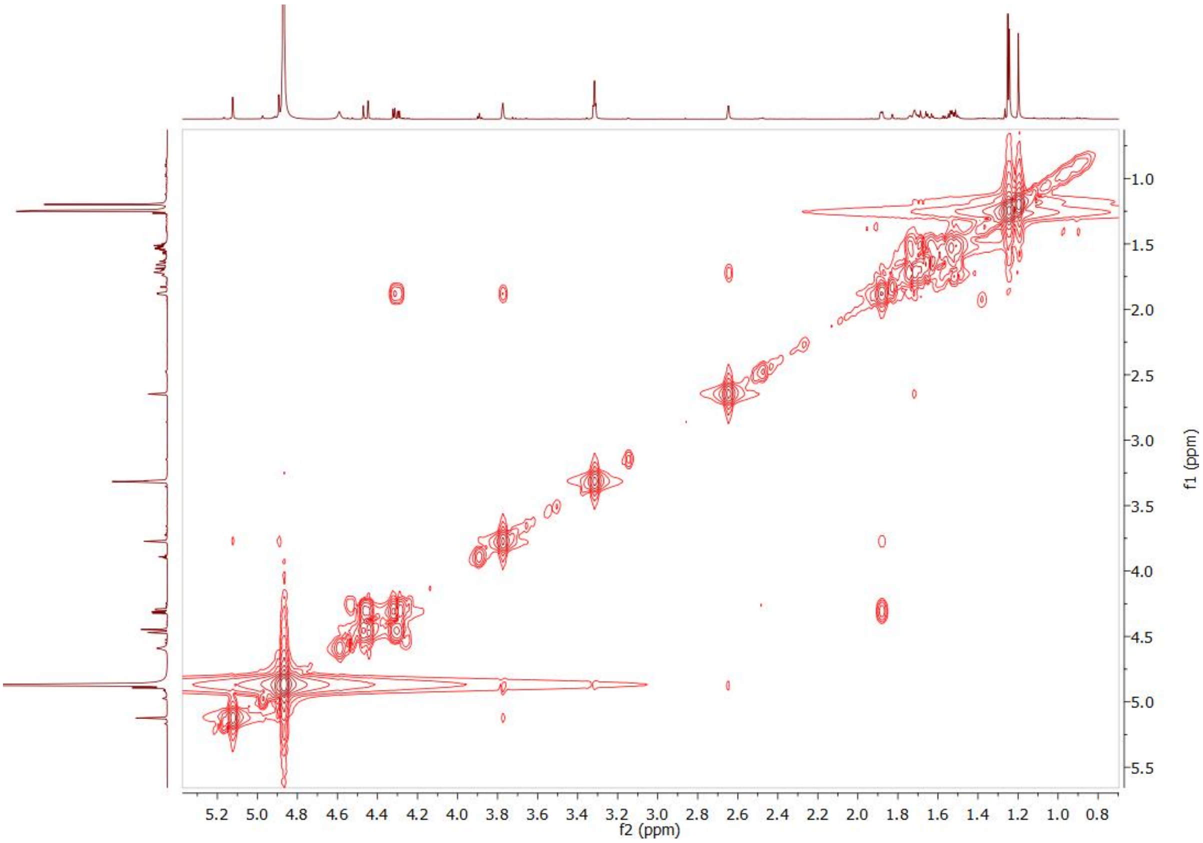

Figure S5: ^{1}H-^{1}H COSY spectrum (500 MHz) of **1** in CD_3OD

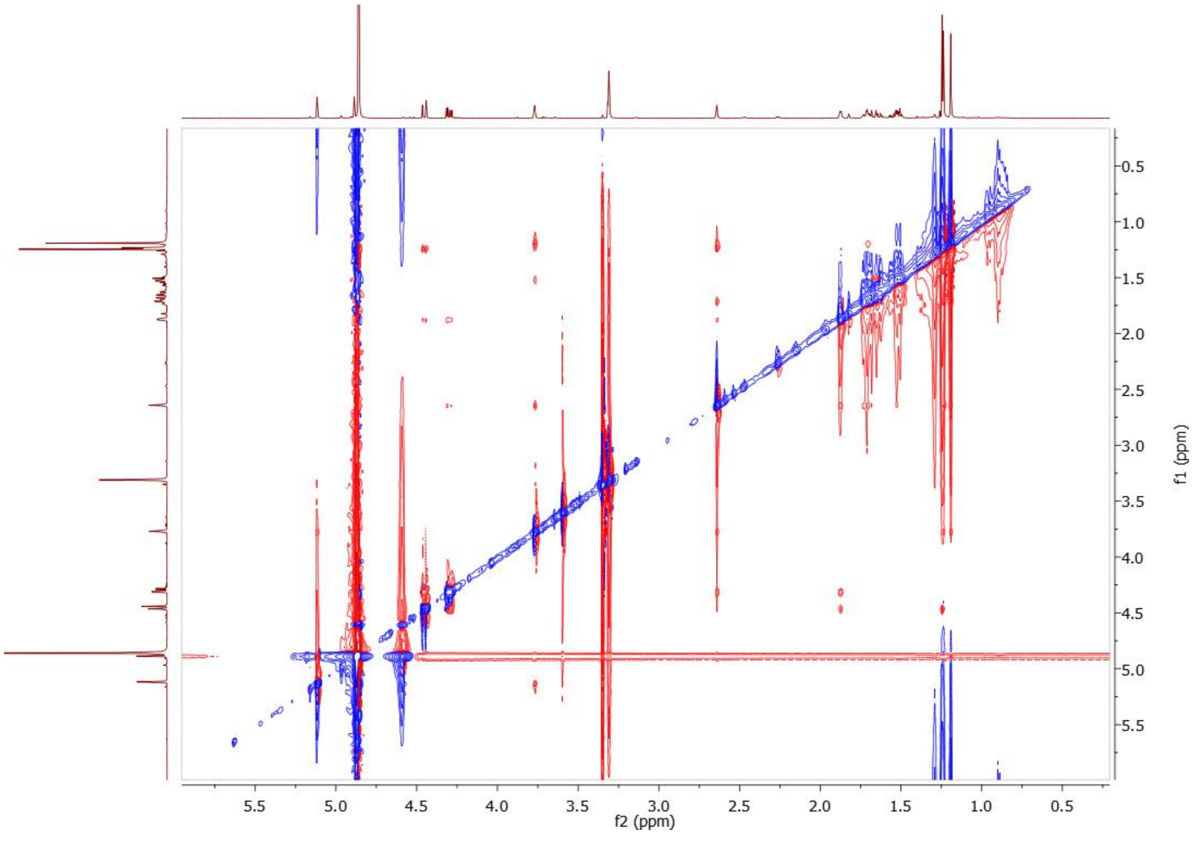

Figure S6: NOESY spectrum (500 MHz) of **1** in CD_3OD

Elemental Composition Report

Single Mass Analysis
Tolerance = 10.0 PPM / DBE: min = -1.5, max = 50.0
Element prediction: Off
Number of isotope peaks used for i-FIT = 3

Monoisotopic Mass, Even Electron Ions
31 formula(e) evaluated with 2 results within limits (up to 50 best isotopic matches for each mass)
Elements Used:
C: 0-20 H: 0-30 O: 0-5 Na: 0-1
ESI+BF12-1729-Jan-2019ESI+BF12-17
ESI+BF12-17
BF12-17+ESI 82 (1.799)

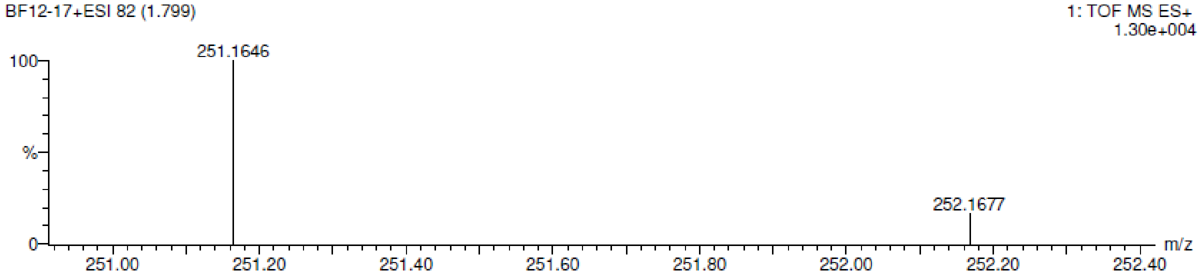

Minimum:				-1.5			
Maximum:		5.0	10.0	50.0			
Mass	Calc. Mass	mDa	PPM	DBE	i-FIT	i-FIT (Norm)	Formula
251.1646	251.1647	-0.1	-0.4	4.5	24.9	0.1	C15 H23 O3
	251.1623	2.3	9.2	1.5	26.8	2.0	C13 H24 O3 Na

Elemental Composition Report

Single Mass Analysis
Tolerance = 10.0 PPM / DBE: min = -1.5, max = 50.0
Element prediction: Off
Number of isotope peaks used for i-FIT = 3

Monoisotopic Mass, Even Electron Ions
32 formula(e) evaluated with 2 results within limits (up to 50 best isotopic matches for each mass)
Elements Used:
C: 0-20 H: 0-30 O: 0-5 Na: 0-1
ESI+BF12-1729-Jan-2019ESI+BF12-17
ESI+BF12-17
BF12-17+ESI 82 (1.799)

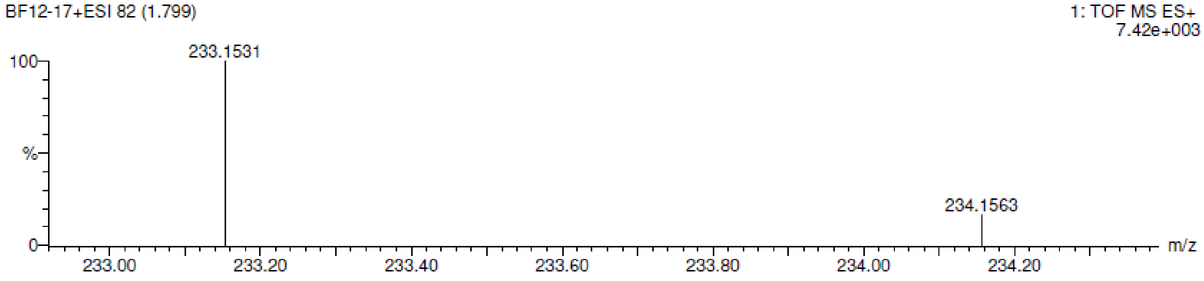

Minimum:				-1.5			
Maximum:		5.0	10.0	50.0			
Mass	Calc. Mass	mDa	PPM	DBE	i-FIT	i-FIT (Norm)	Formula
233.1531	233.1517	1.4	6.0	2.5	23.4	0.6	C13 H22 O2 Na
	233.1542	-1.1	-4.7	5.5	23.5	0.8	C15 H21 O2

Figure S7: HRESIMS spectrum of **1**

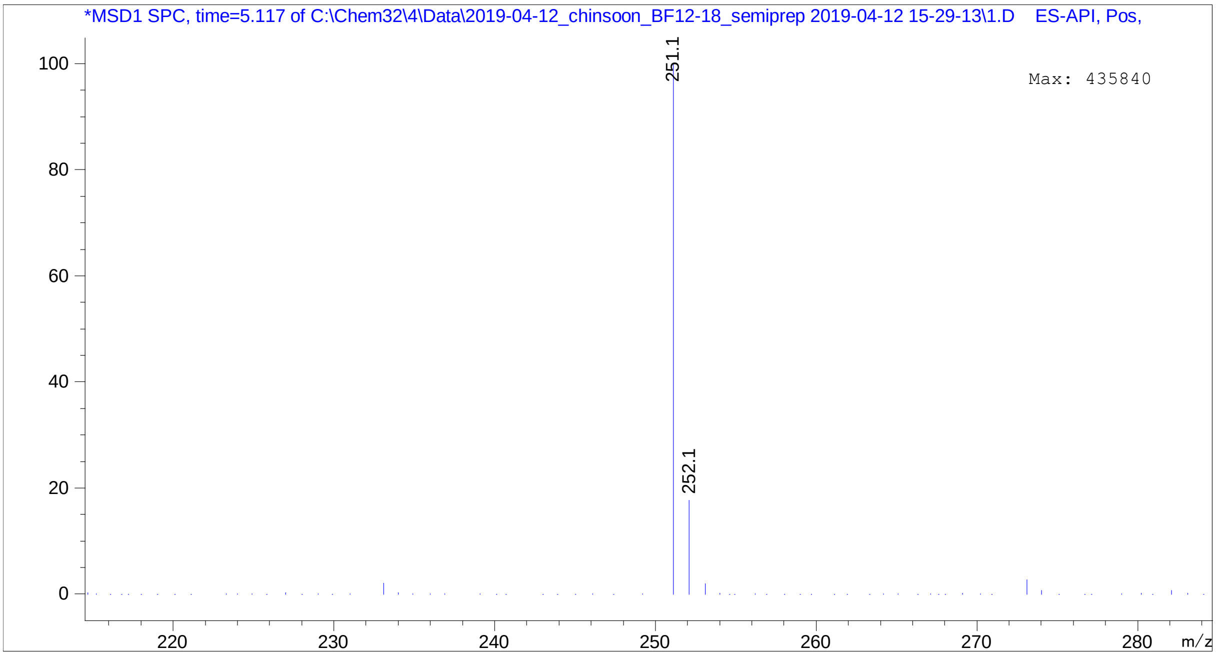

Figure S8: ESIMS spectrum of **1**

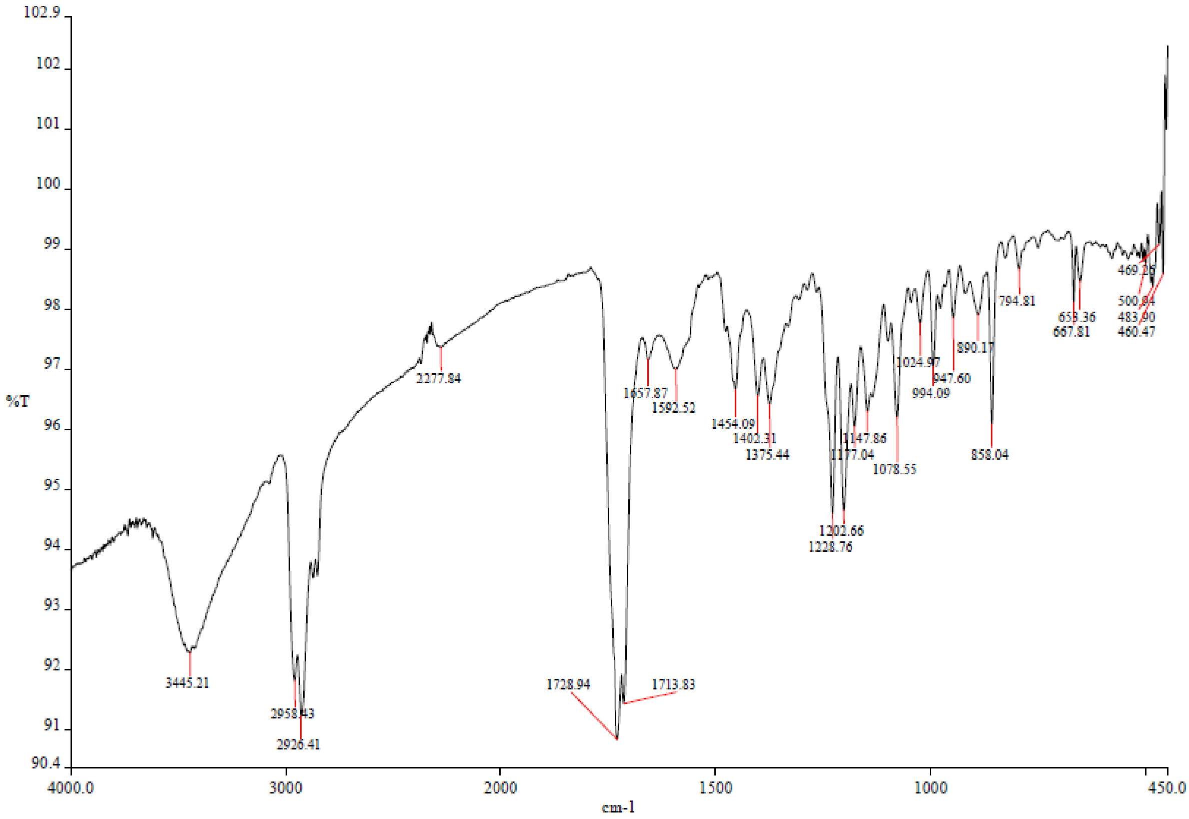

Figure S9: IR spectrum of **1**

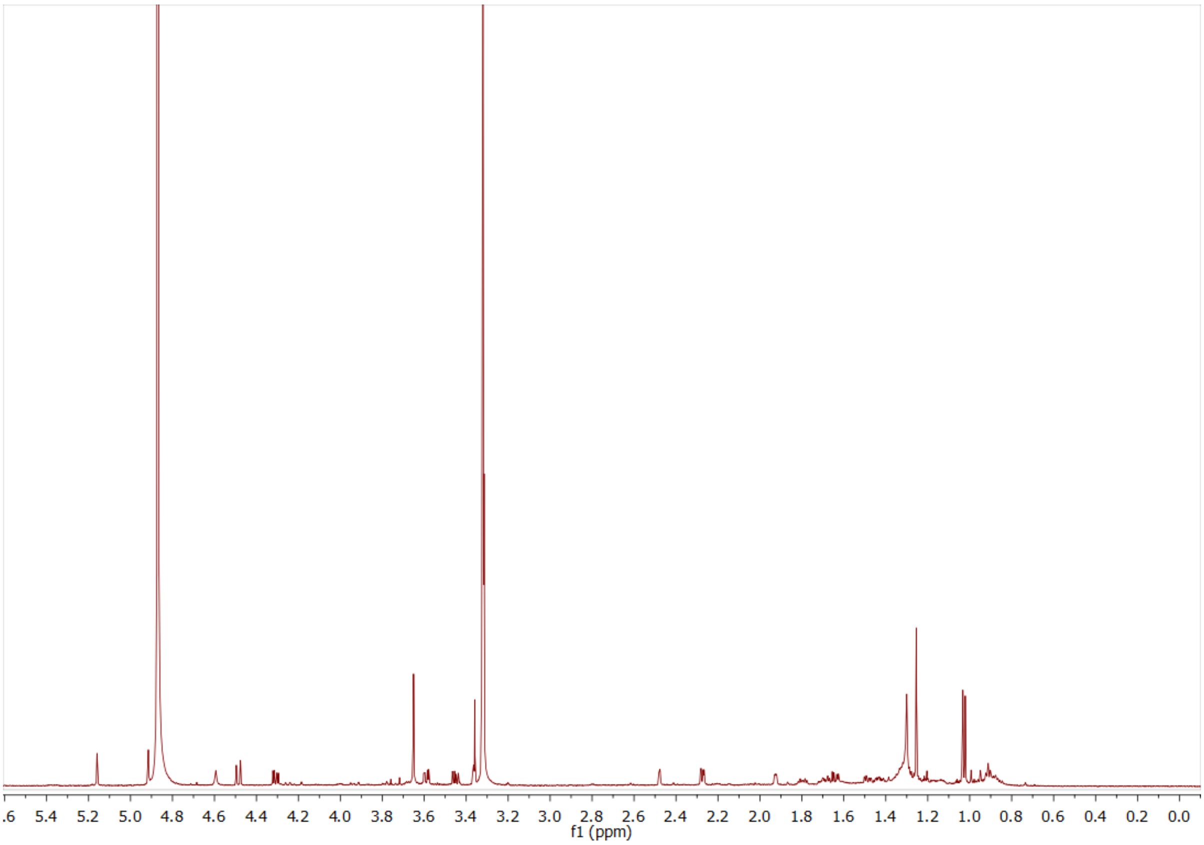

Figure S10: ^{1}H NMR spectrum (600 MHz) of **2** in CD_3OD

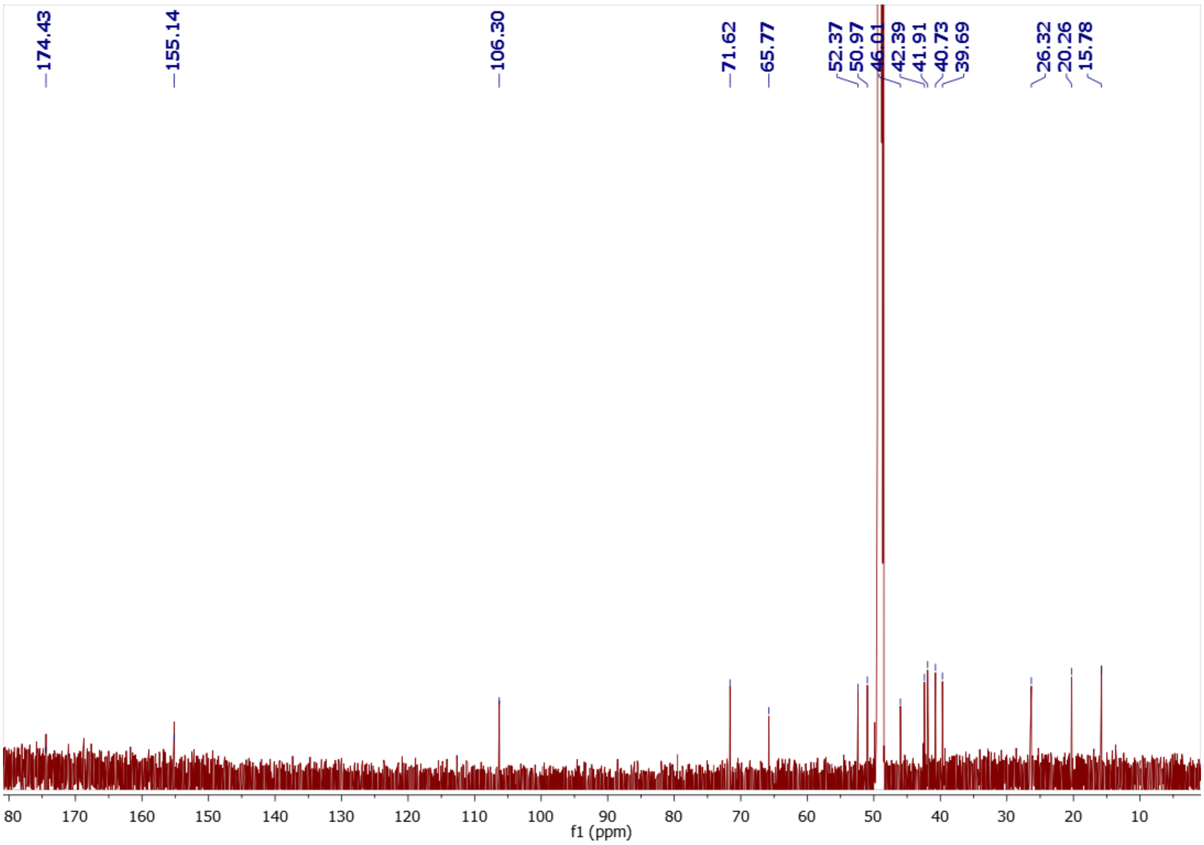

Figure S11: ^{13}C NMR spectrum (150 MHz) of **2** in CD_3OD

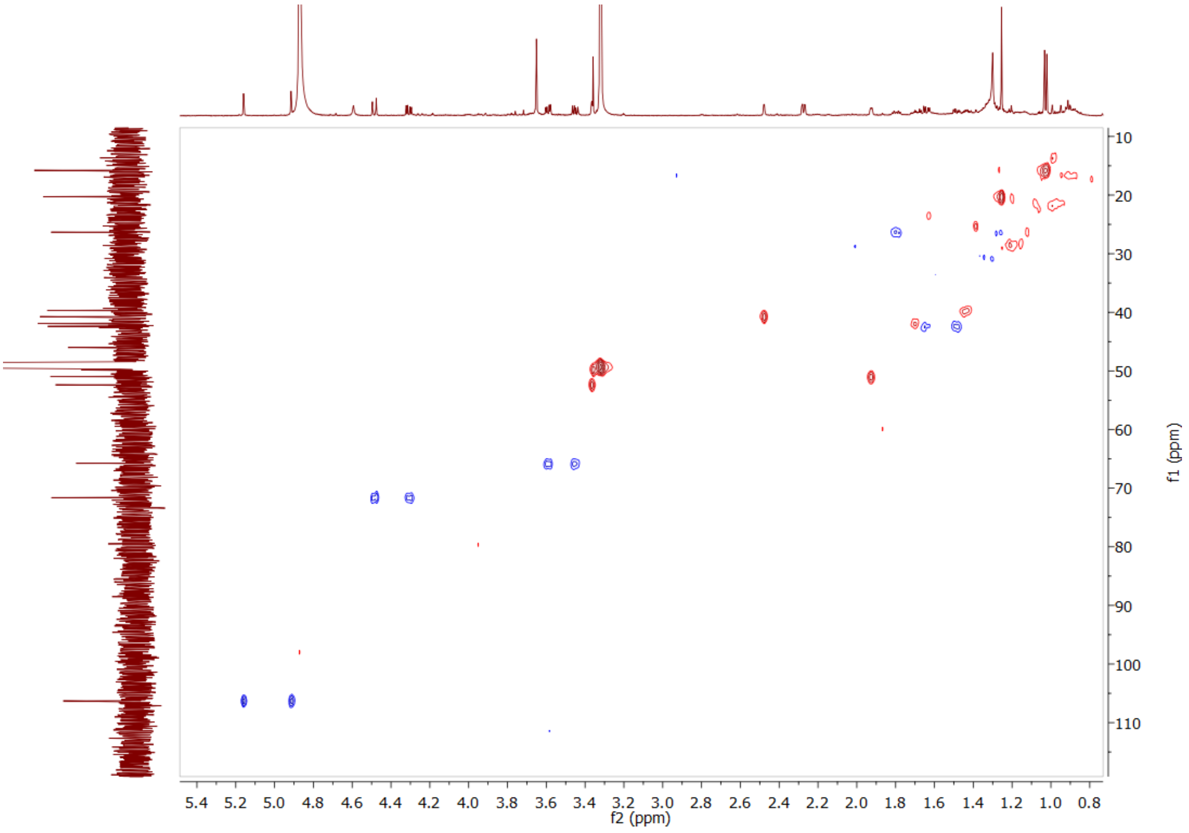

Figure S12: HSQC spectrum (600 MHz) of **2** in CD_3OD

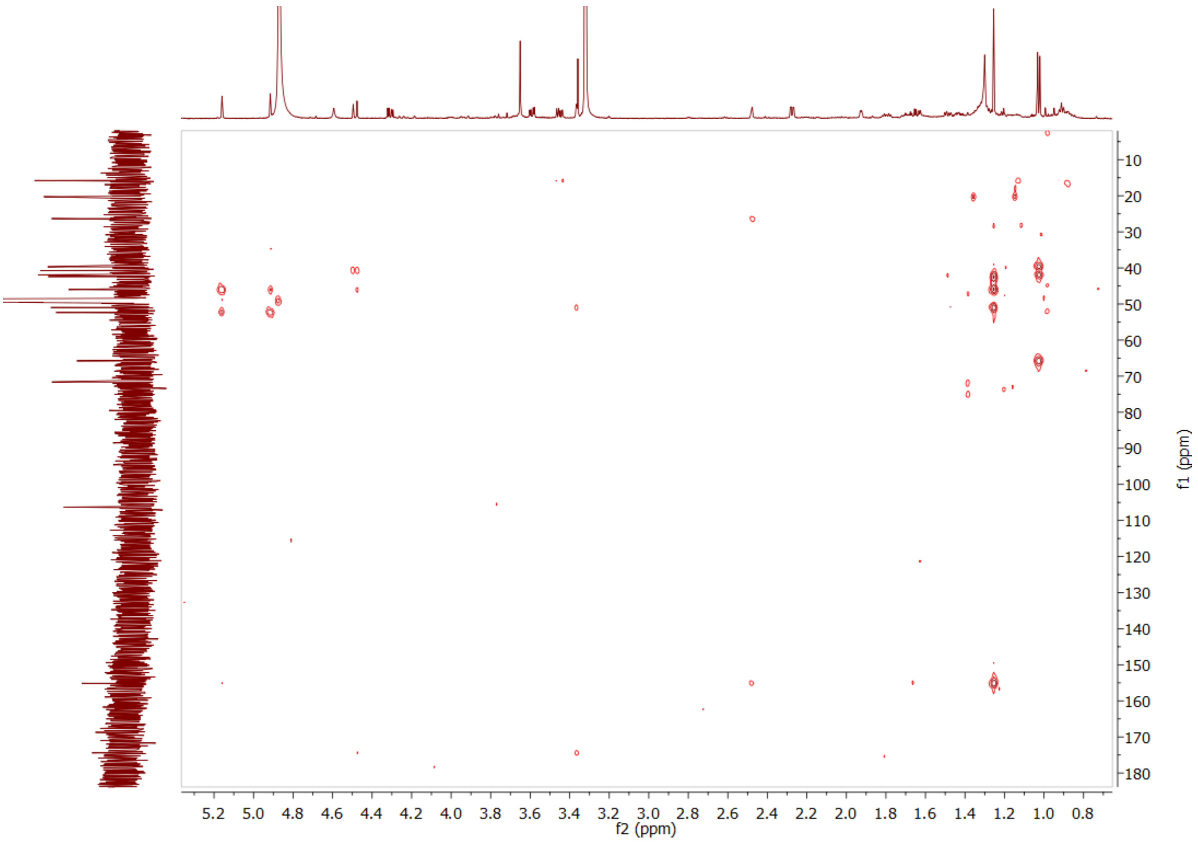

Figure S13: HMBC spectrum (600 MHz) of **2** in CD_3OD

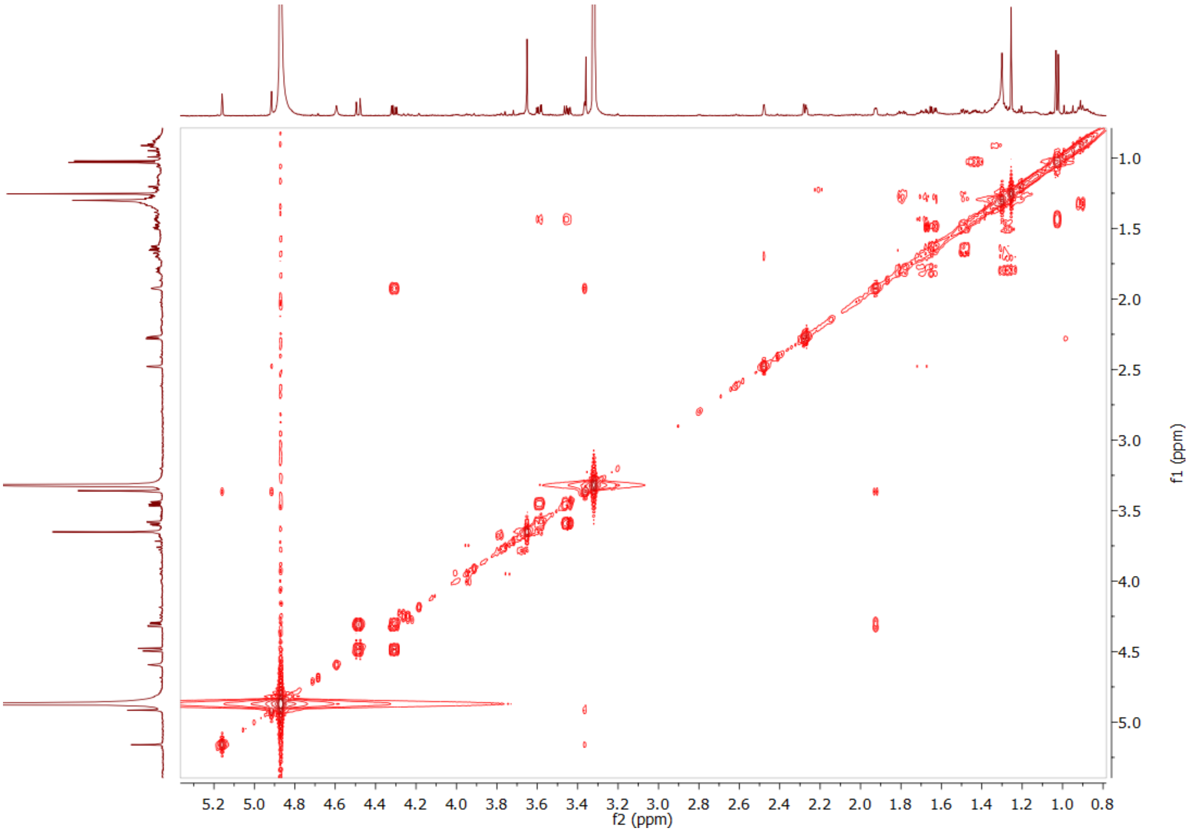

Figure S14: ^{1}H-^{1}H COSY spectrum (600 MHz) of **2** in CD_3OD

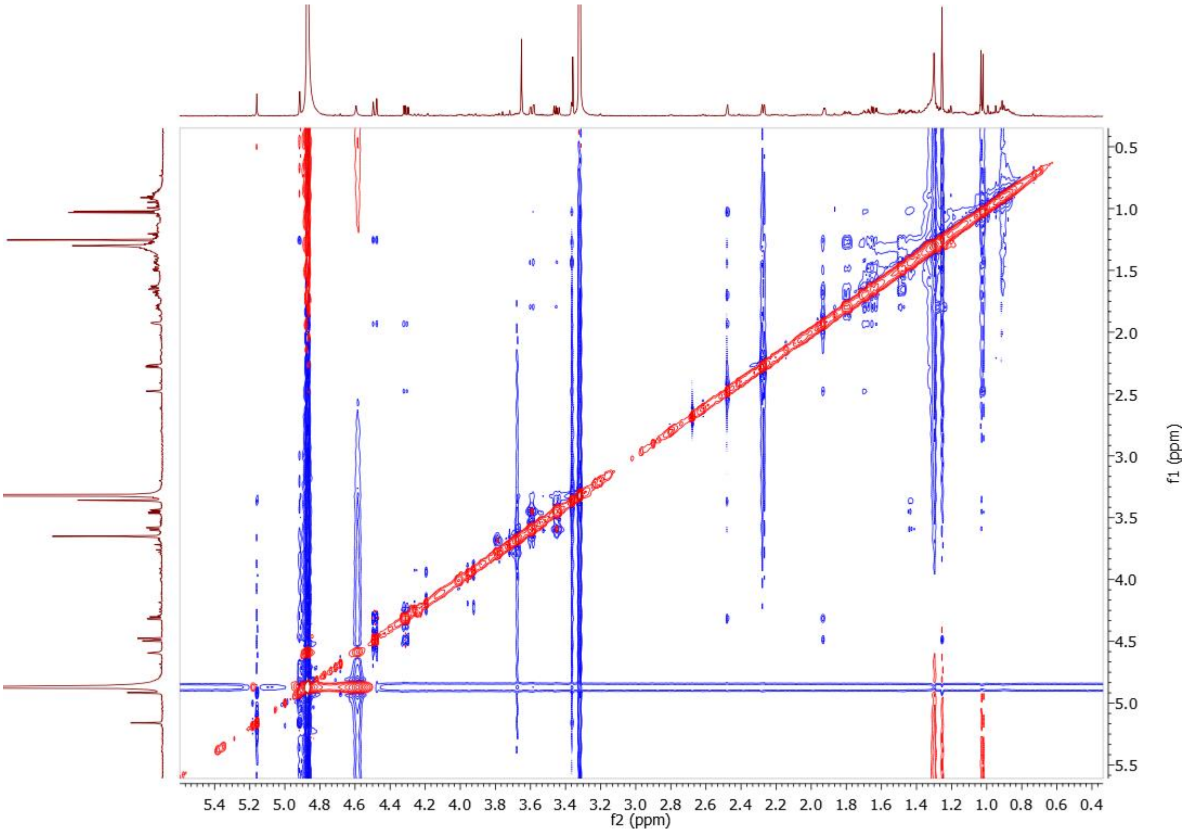

Figure S15: NOESY spectrum (600 MHz) of **2** in CD_3OD

Elemental Composition Report

Single Mass Analysis
Tolerance = 10.0 PPM / DBE: min = -1.5, max = 50.0
Element prediction: Off
Number of isotope peaks used for i-FIT = 3

Monoisotopic Mass, Even Electron Ions
35 formula(e) evaluated with 1 results within limits (up to 50 best isotopic matches for each mass)
Elements Used:
C: 0-50 H: 0-50 O: 0-10
BF12-1415-Apr-2019BF12-14
BF12-14
BF12-14 9 (0.195)
1: TOF MS ES+
2.16e+003

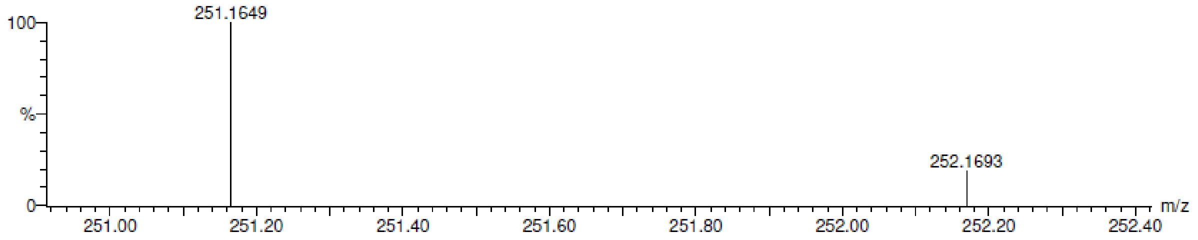

Minimum:				-1.5			
Maximum:		5.0	10.0	50.0			
Mass	Calc. Mass	mDa	PPM	DBE	i-FIT	i-FIT (Norm)	Formula
251.1649	251.1647	0.2	0.8	4.5	19.6	0.0	C15 H23 O3

Elemental Composition Report

Single Mass Analysis
Tolerance = 10.0 PPM / DBE: min = -1.5, max = 50.0
Element prediction: Off
Number of isotope peaks used for i-FIT = 3

Monoisotopic Mass, Even Electron Ions
31 formula(e) evaluated with 1 results within limits (up to 50 best isotopic matches for each mass)
Elements Used:
C: 0-50 H: 0-50 O: 0-10
BF12-1415-Apr-2019BF12-14
BF12-14
BF12-14 9 (0.195)
1: TOF MS ES+
6.97e+002

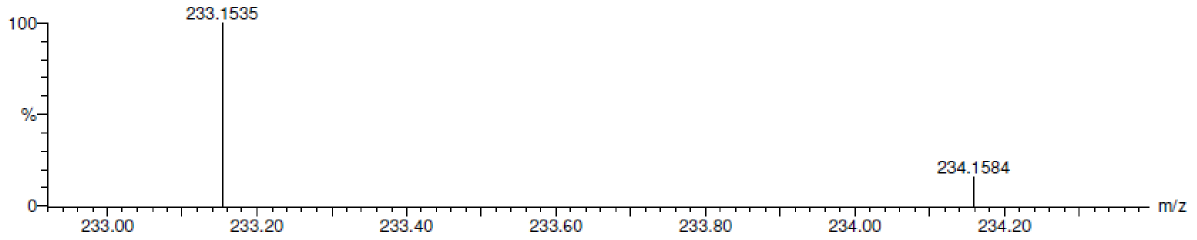

Minimum:				-1.5			
Maximum:		5.0	10.0	50.0			
Mass	Calc. Mass	mDa	PPM	DBE	i-FIT	i-FIT (Norm)	Formula
233.1535	233.1542	-0.7	-3.0	5.5	14.8	0.0	C15 H21 O2

Figure S16: HRESIMS spectrum of **2**

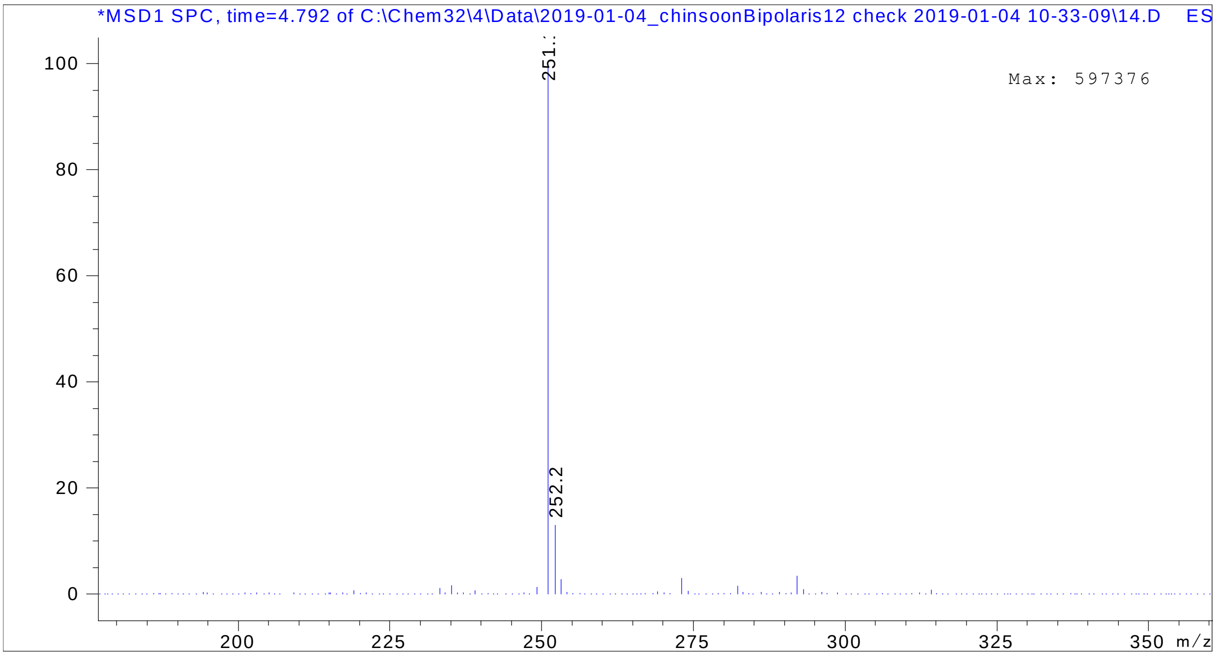

Figure S17: ESIMS spectrum of **2**

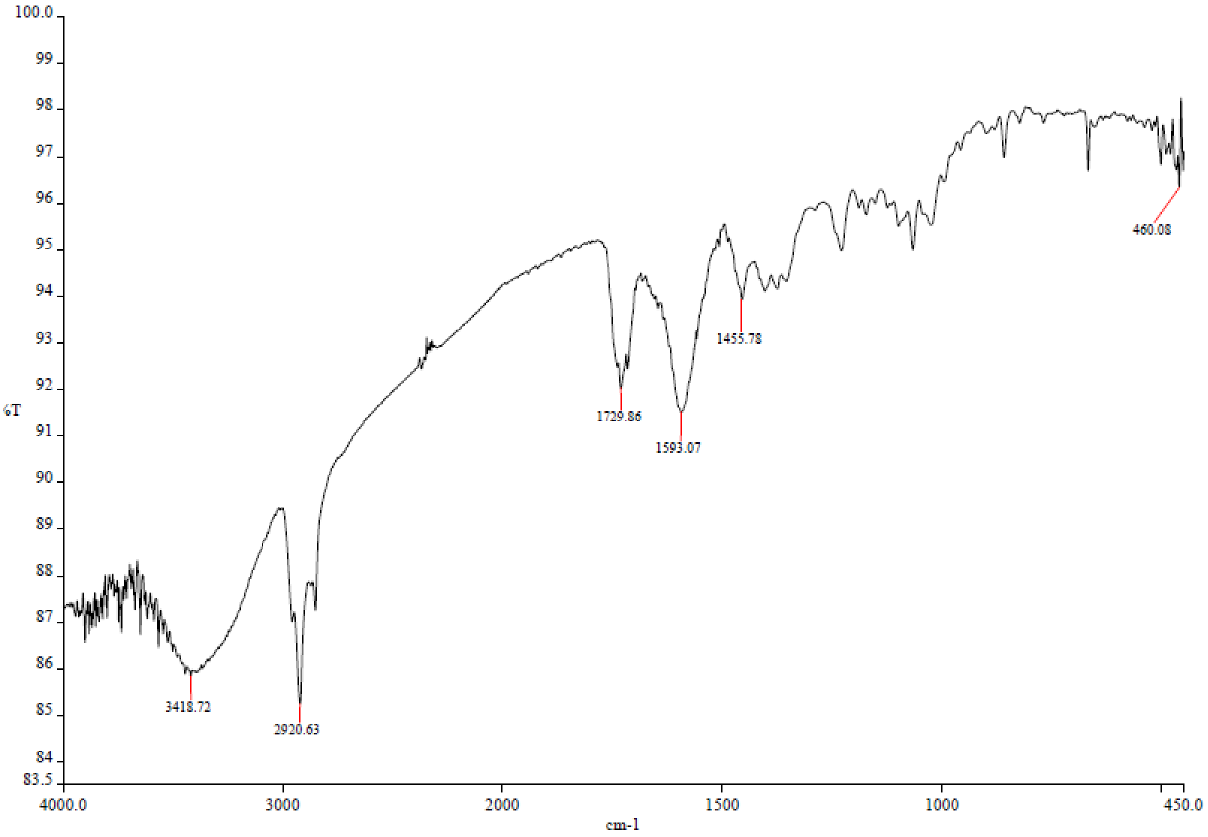

Figure S18: IR spectrum of **2**

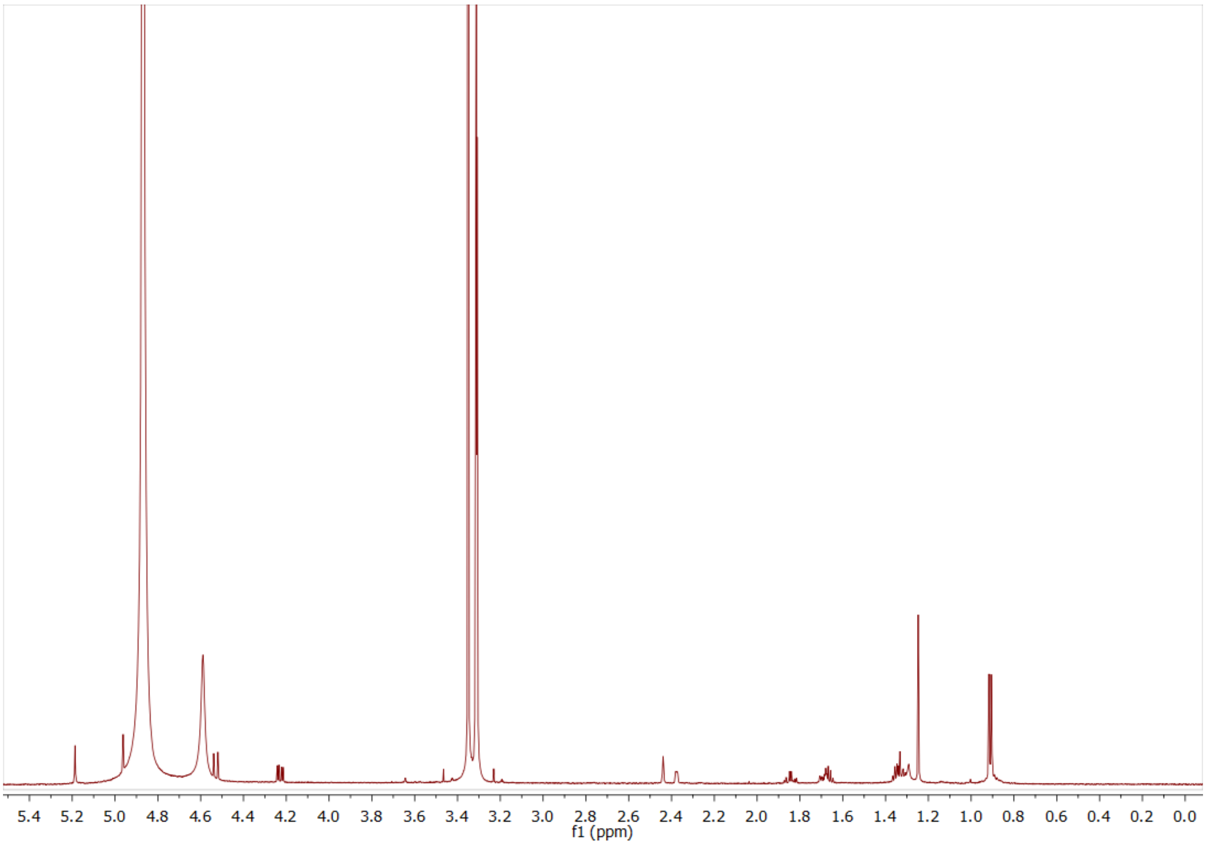

Figure S19: ^{1}H NMR spectrum (600 MHz) of **3** in CD_3OD

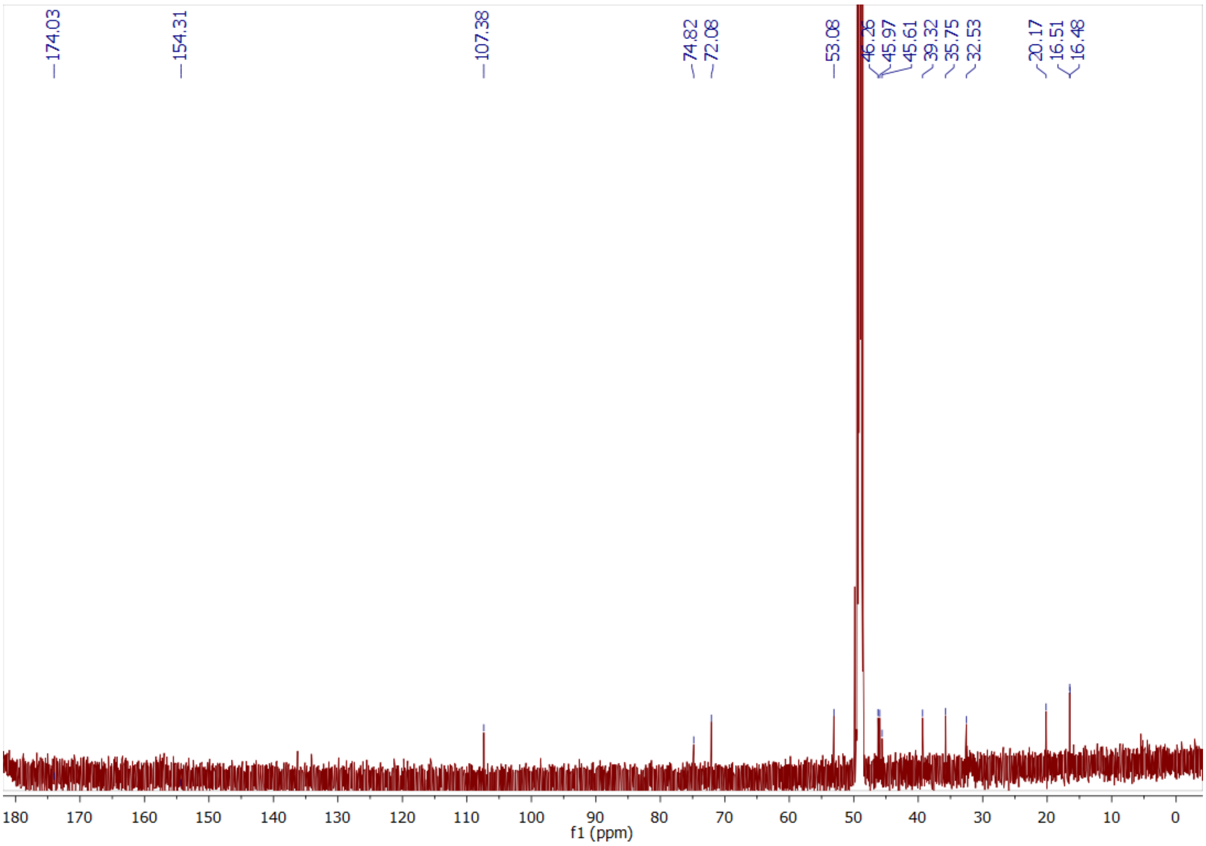

Figure S20: ^{13}C NMR spectrum (150 MHz) of **3** in CD_3OD

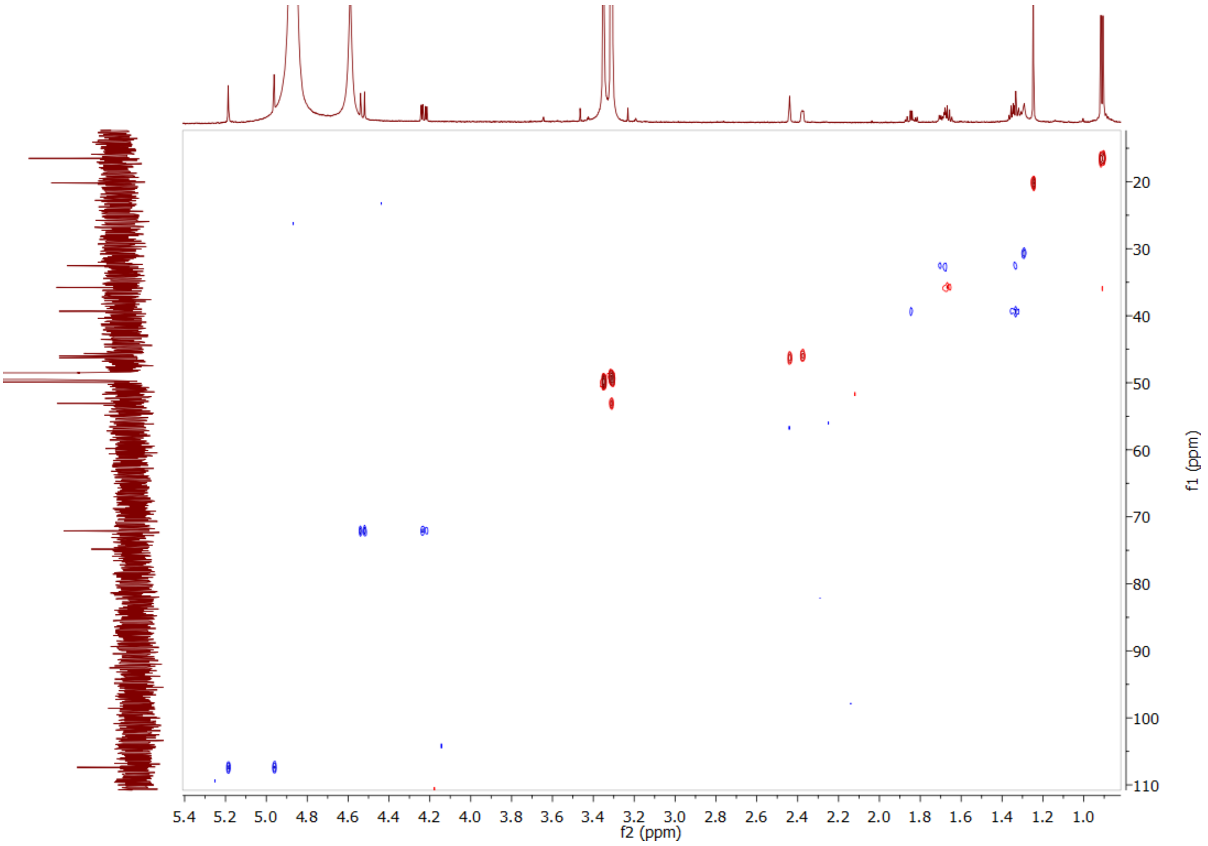

Figure S21: HSQC spectrum (600 MHz) of **3** in CD_3OD

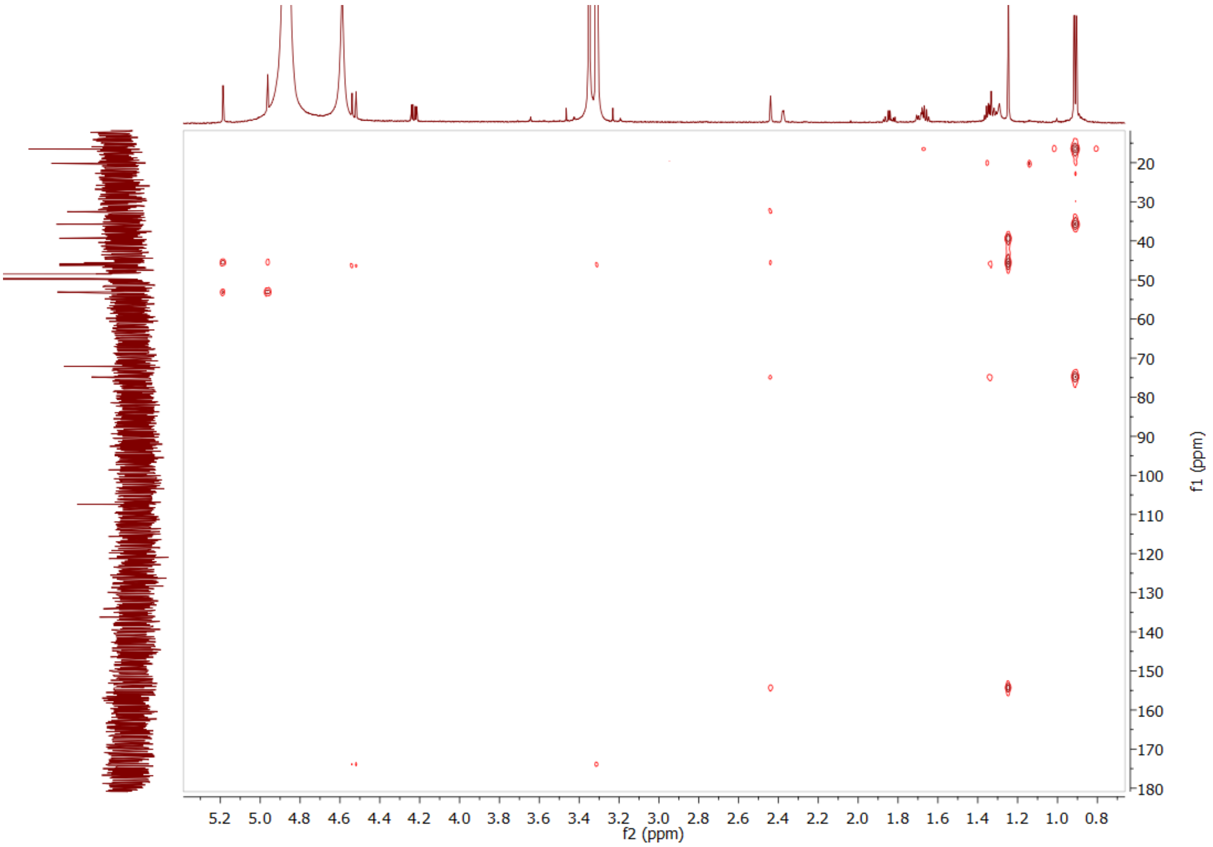

Figure S22: HMBC spectrum (600 MHz) of **3** in CD_3OD

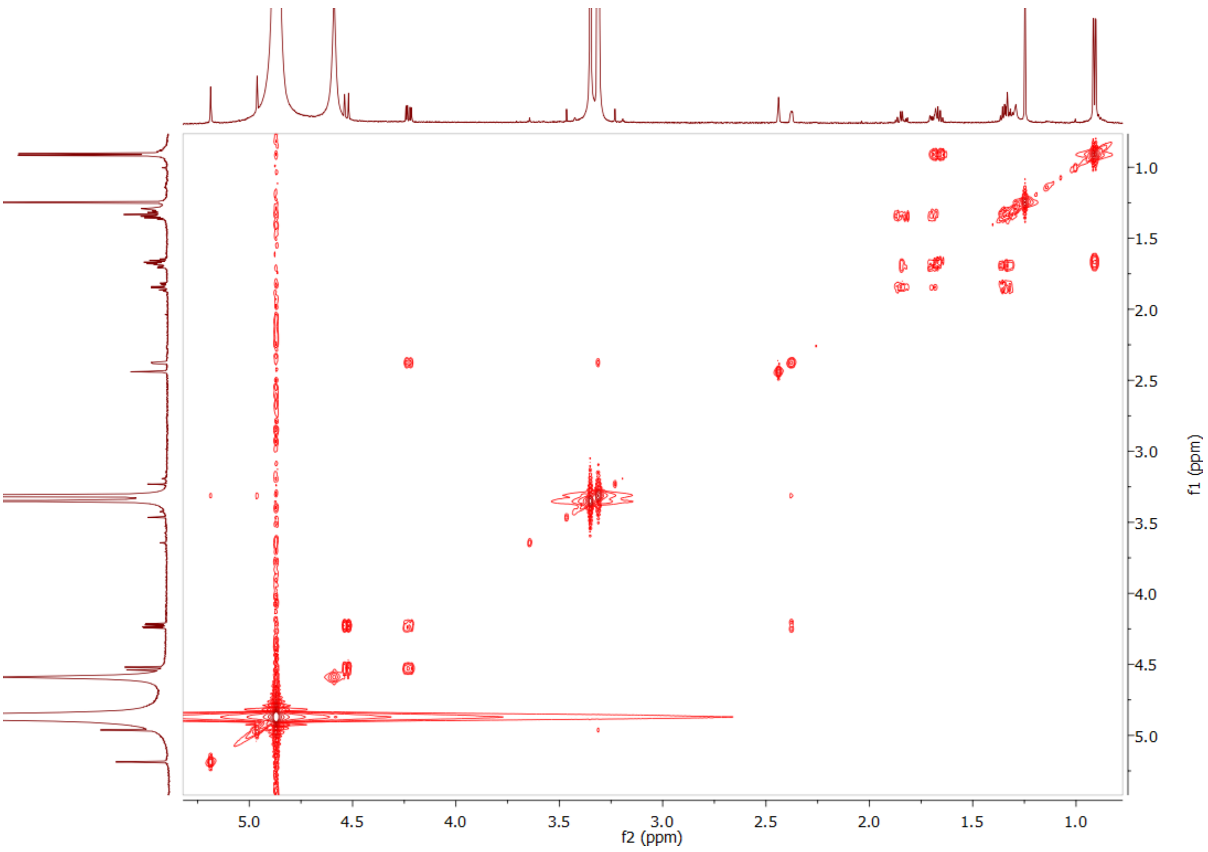

Figure S23: ^{1}H-^{1}H COSY spectrum (600 MHz) of **3** in CD_3OD

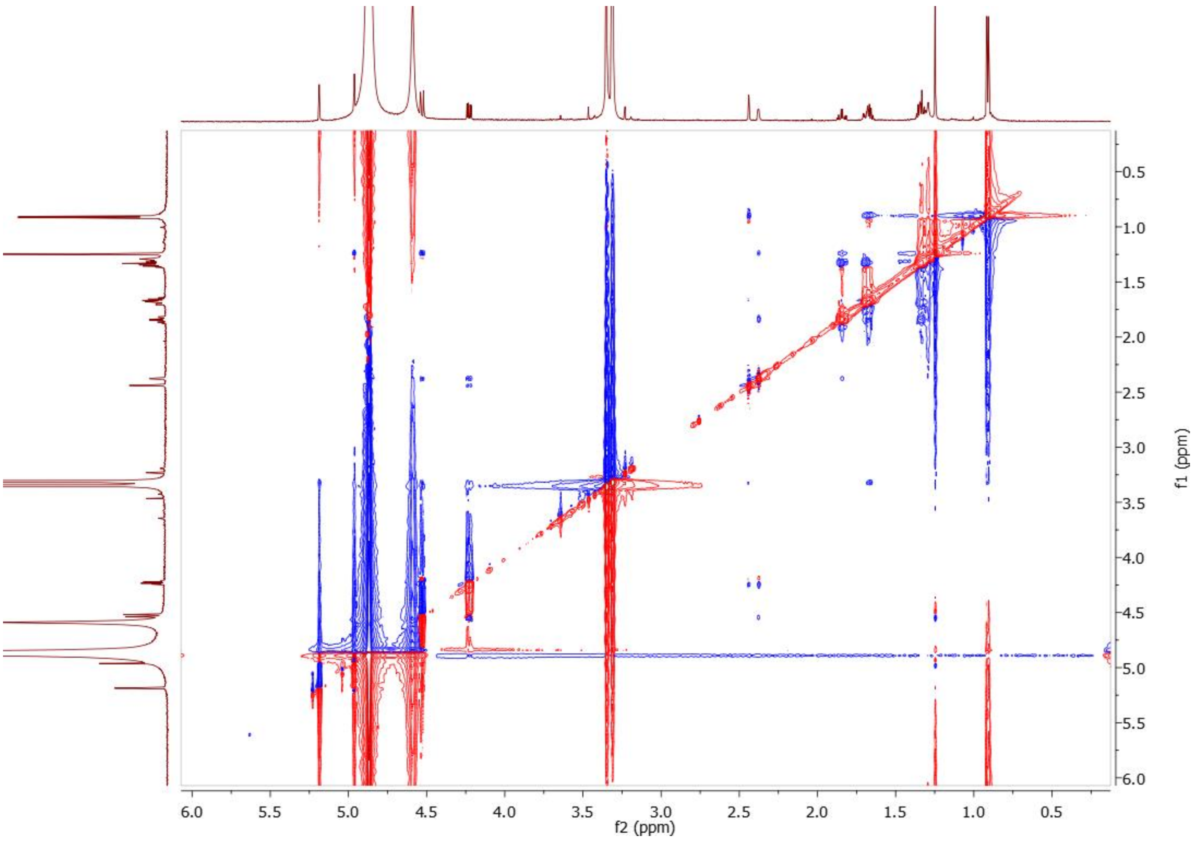

Figure S24: NOESY spectrum (600 MHz) of **3** in CD_3OD

Elemental Composition Report

Single Mass Analysis
Tolerance = 10.0 PPM / DBE: min = -1.5, max = 50.0
Element prediction: Off
Number of isotope peaks used for i-FIT = 3

Monoisotopic Mass, Even Electron Ions
35 formula(e) evaluated with 1 results within limits (up to 50 best isotopic matches for each mass)
Elements Used:
C: 0-50 H: 0-50 O: 0-10
BF12-8-215-Apr-2019BF12-8-2
BF12-8-2
BF12-8-2 9 (0.195)

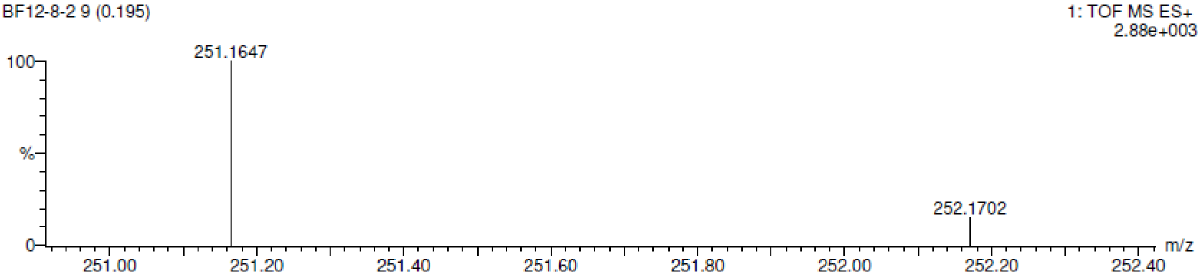

Minimum:				-1.5			
Maximum:		5.0	10.0	50.0			
Mass	Calc. Mass	mDa	PPM	DBE	i-FIT	i-FIT (Norm)	Formula
251.1647	251.1647	0.0	0.0	4.5	22.2	0.0	C15 H23 O3

Elemental Composition Report

Single Mass Analysis
Tolerance = 10.0 PPM / DBE: min = -1.5, max = 50.0
Element prediction: Off
Number of isotope peaks used for i-FIT = 3

Monoisotopic Mass, Even Electron Ions
31 formula(e) evaluated with 1 results within limits (up to 50 best isotopic matches for each mass)
Elements Used:
C: 0-50 H: 0-50 O: 0-10
BF12-8-215-Apr-2019BF12-8-2
BF12-8-2
BF12-8-2 10 (0.212)

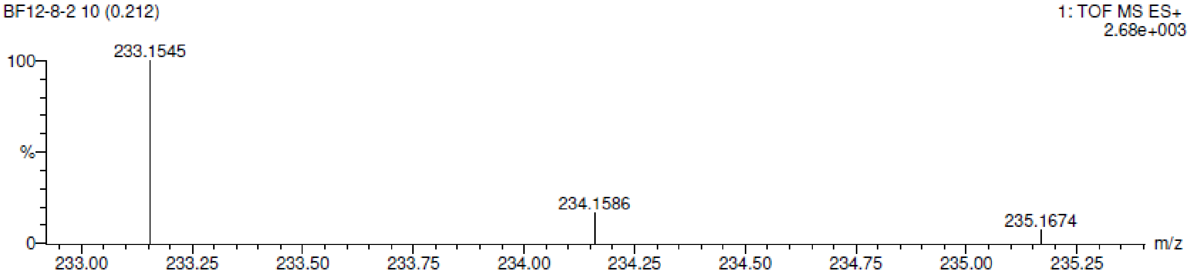

Minimum:				-1.5			
Maximum:		5.0	10.0	50.0			
Mass	Calc. Mass	mDa	PPM	DBE	i-FIT	i-FIT (Norm)	Formula
233.1545	233.1542	0.3	1.3	5.5	16.9	0.0	C15 H21 O2

Figure S25: HRESIMS spectrum of **3**

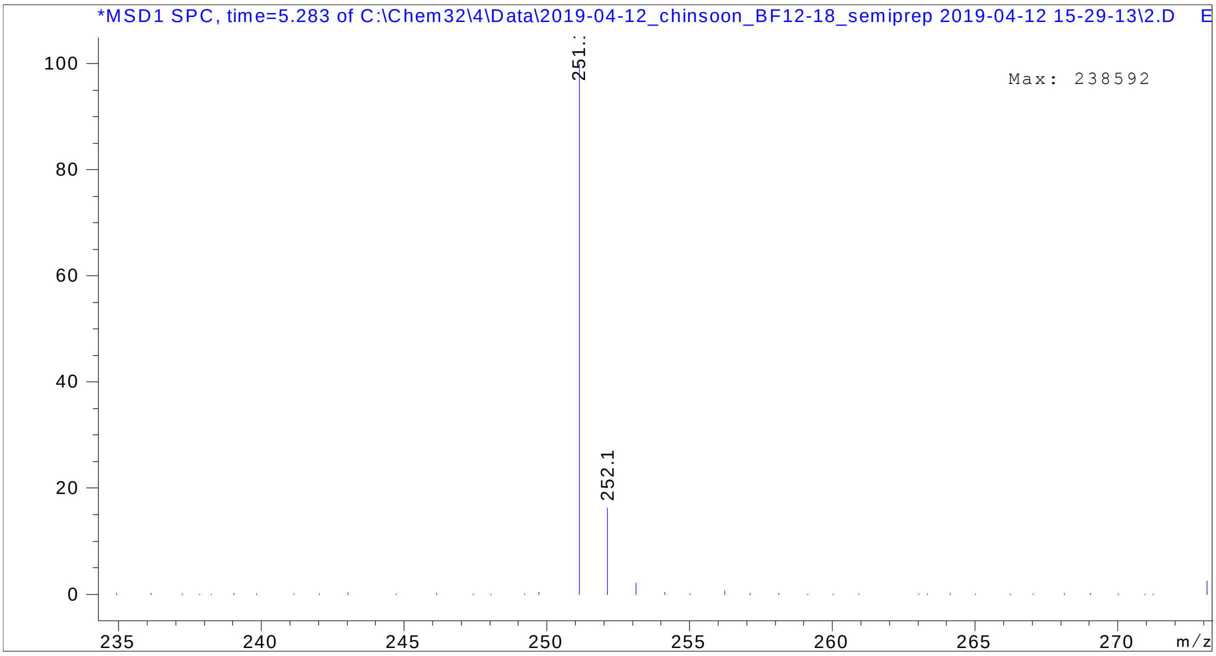

Figure S26: ESIMS spectrum of **3**

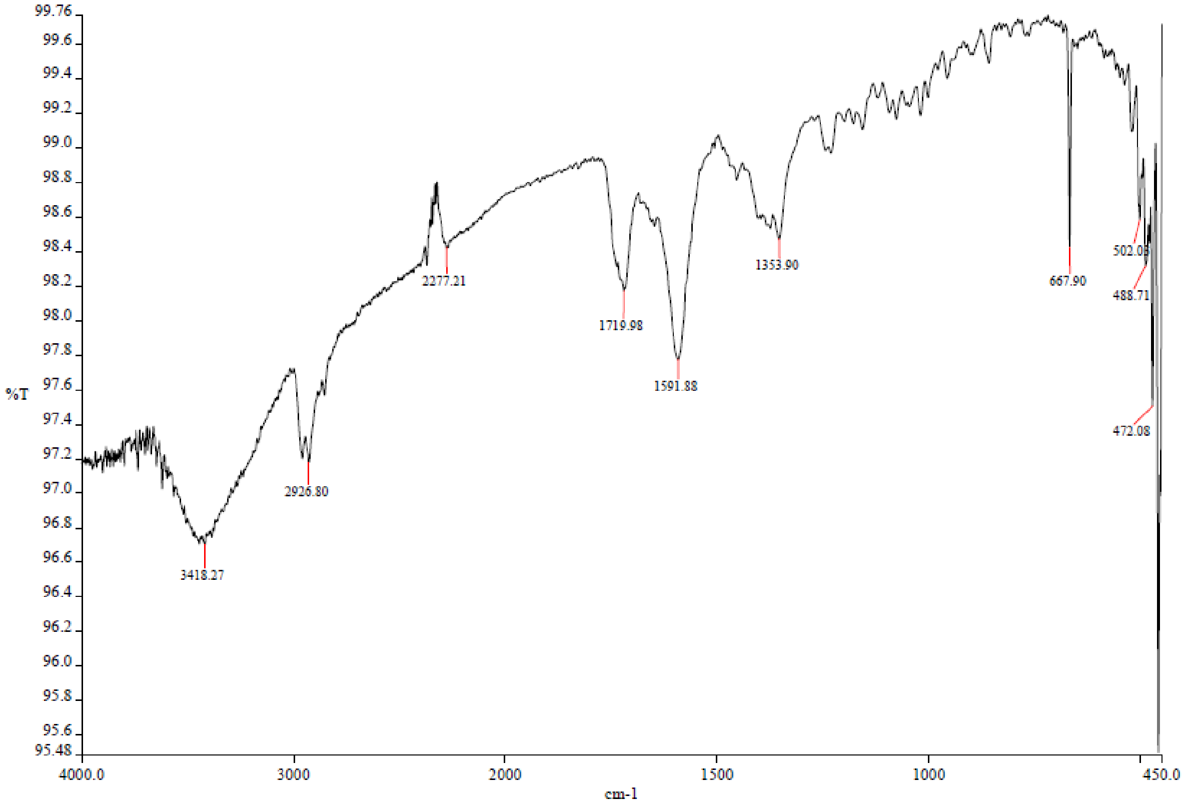

Figure S27: IR spectrum of **3**

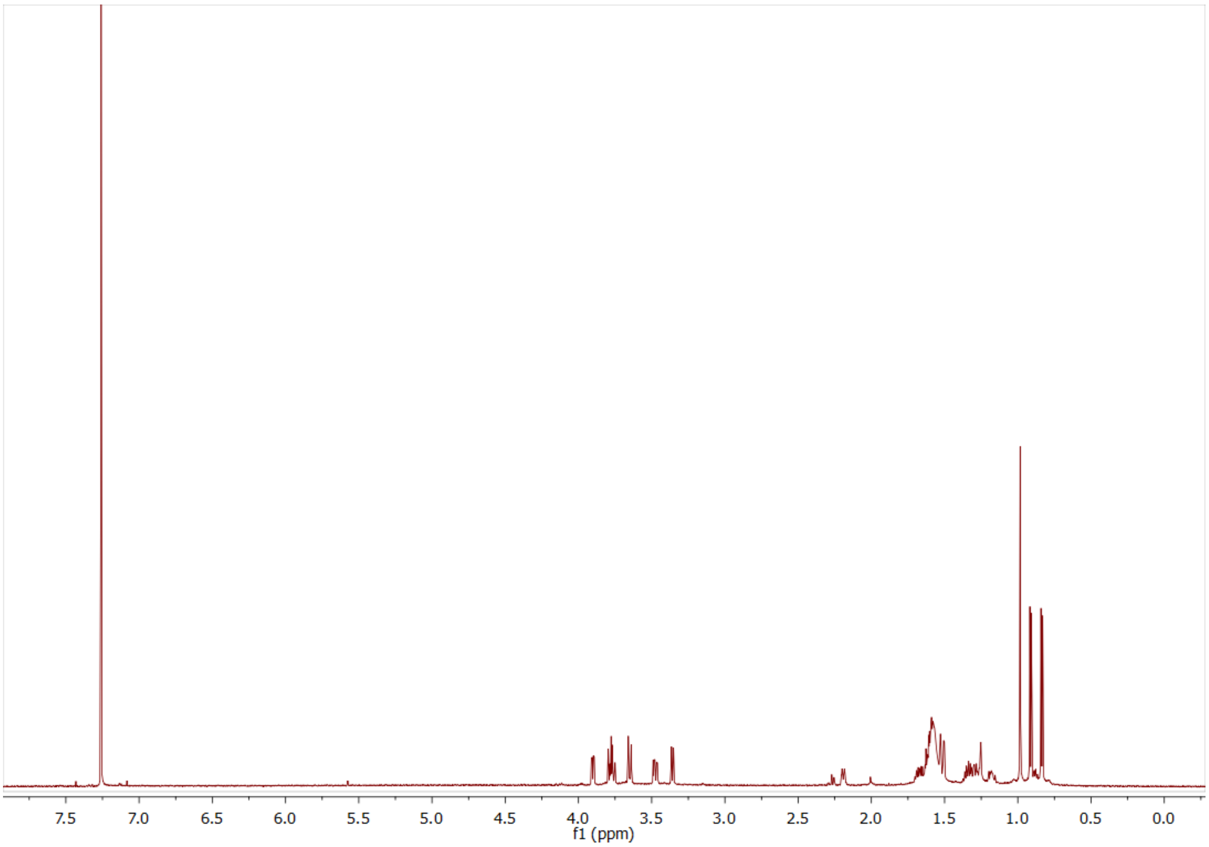

Figure S28: ^{1}H NMR spectrum (600 MHz) of **4** in $CDCl_3$

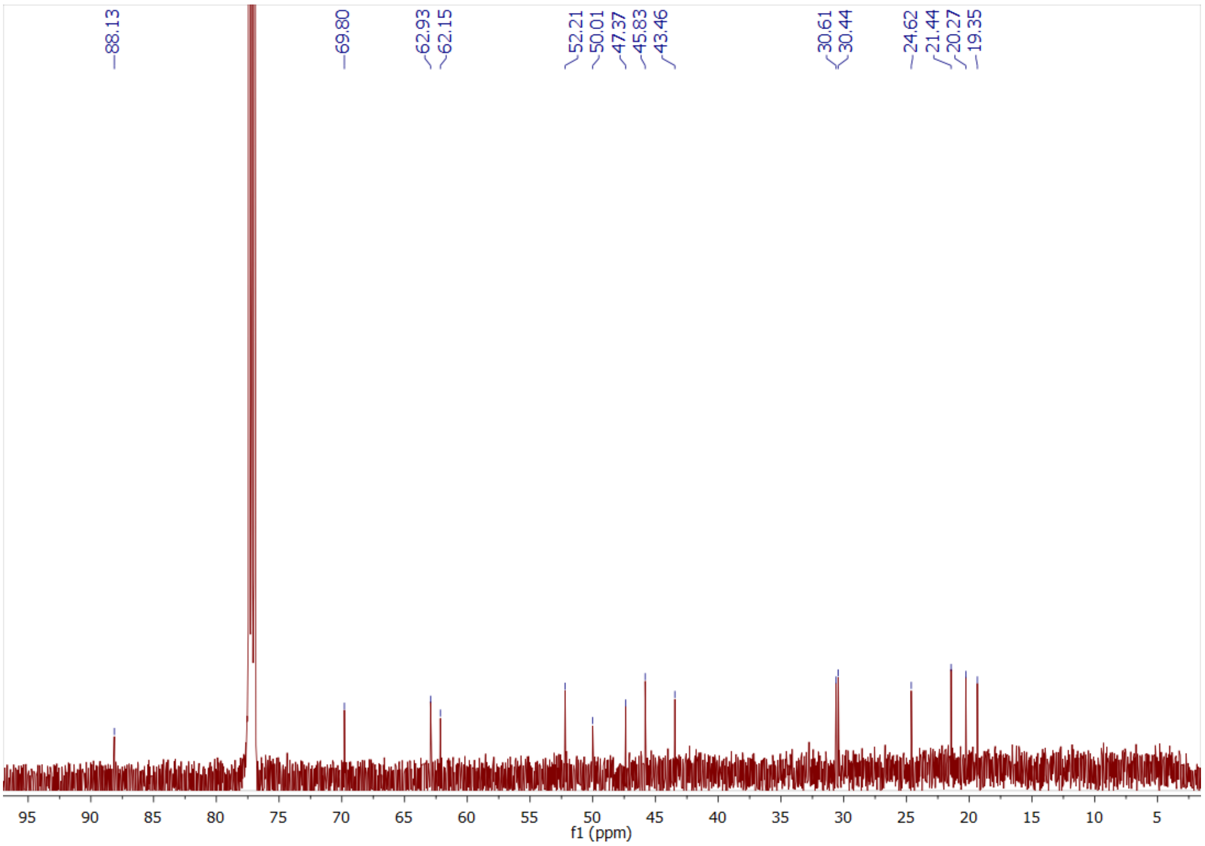

Figure S29: ^{13}C NMR spectrum (150 MHz) of **4** in $CDCl_3$

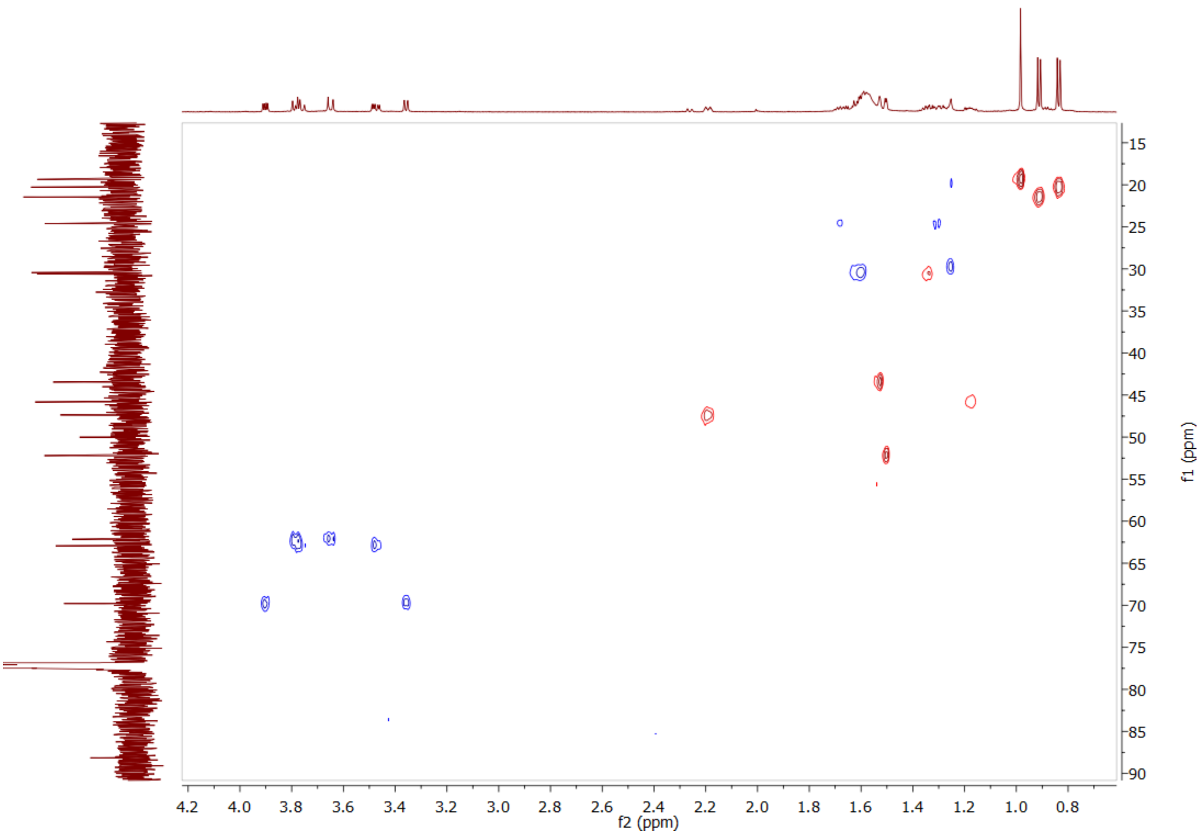

Figure S30: HSQC spectrum (600 MHz) of **4** in $CDCl_3$

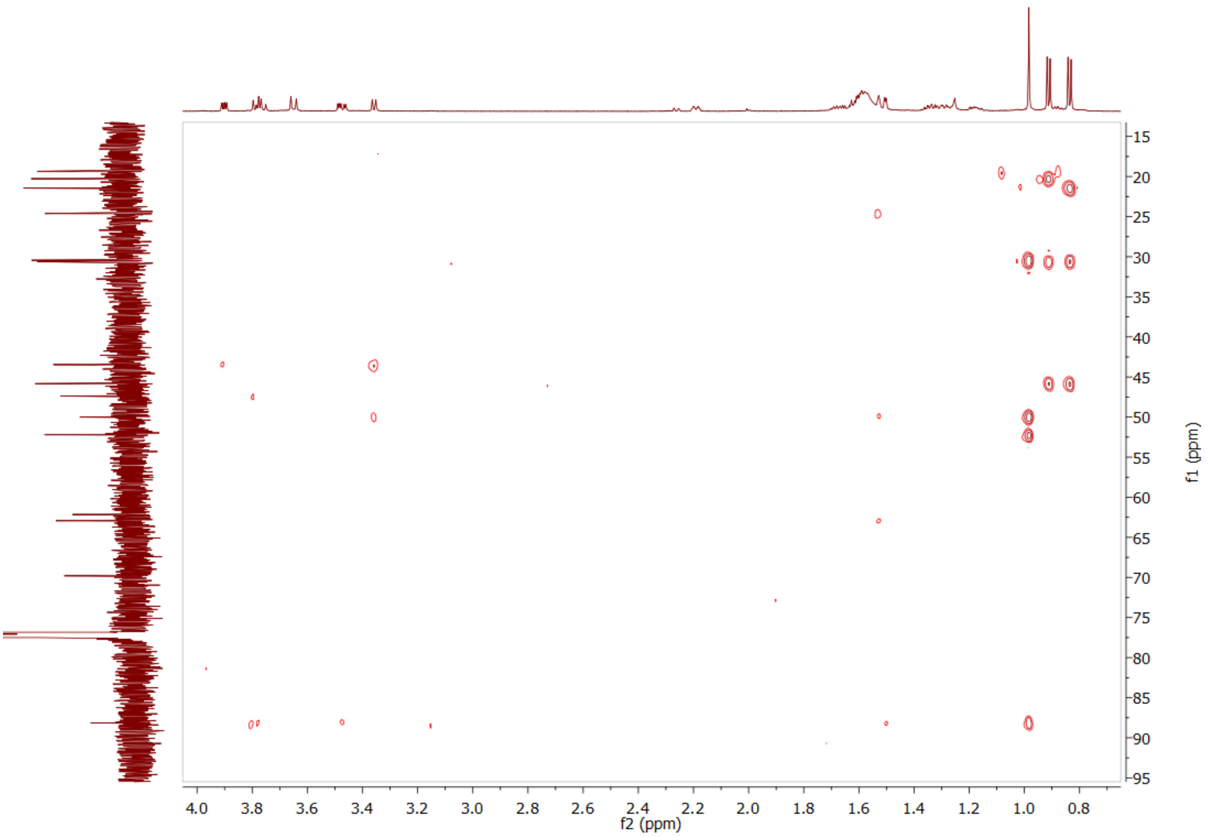

Figure S31: HMBC spectrum (600 MHz) of **4** in $CDCl_3$

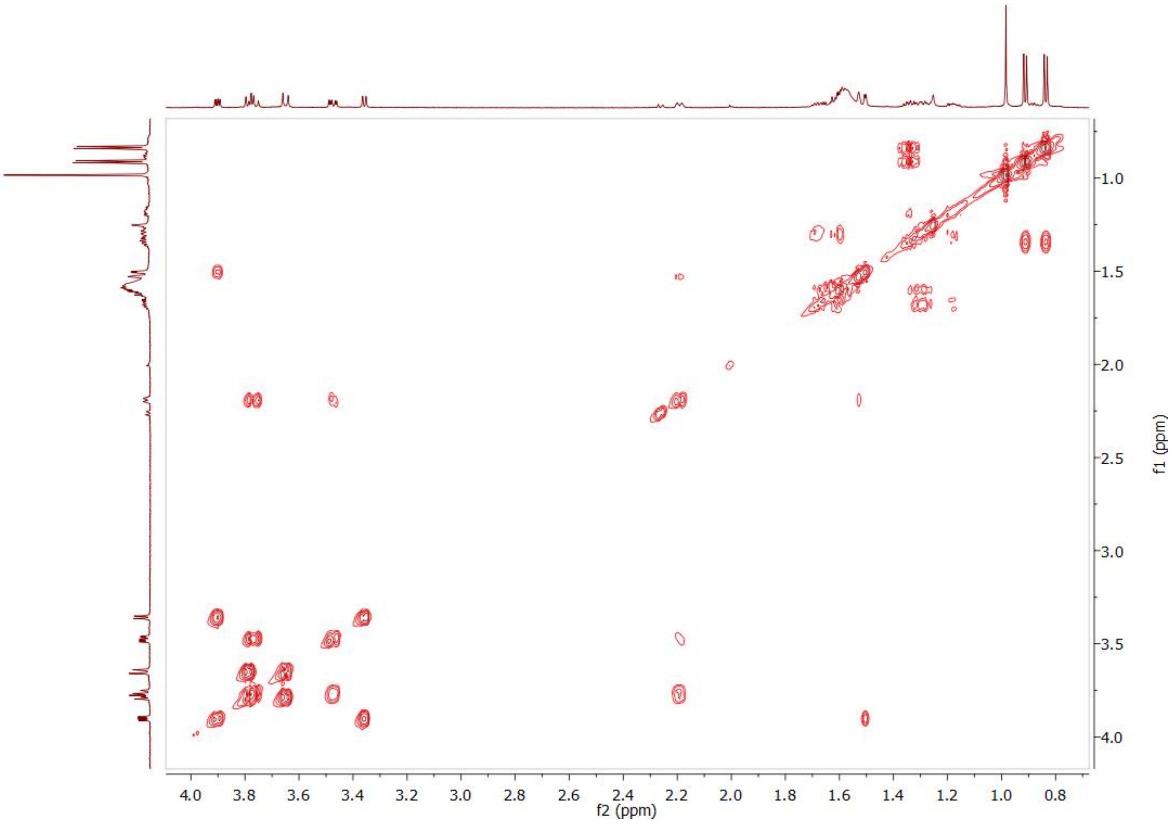

Figure S32: ^{1}H-^{1}H COSY spectrum (600 MHz) of **4** in $CDCl_3$

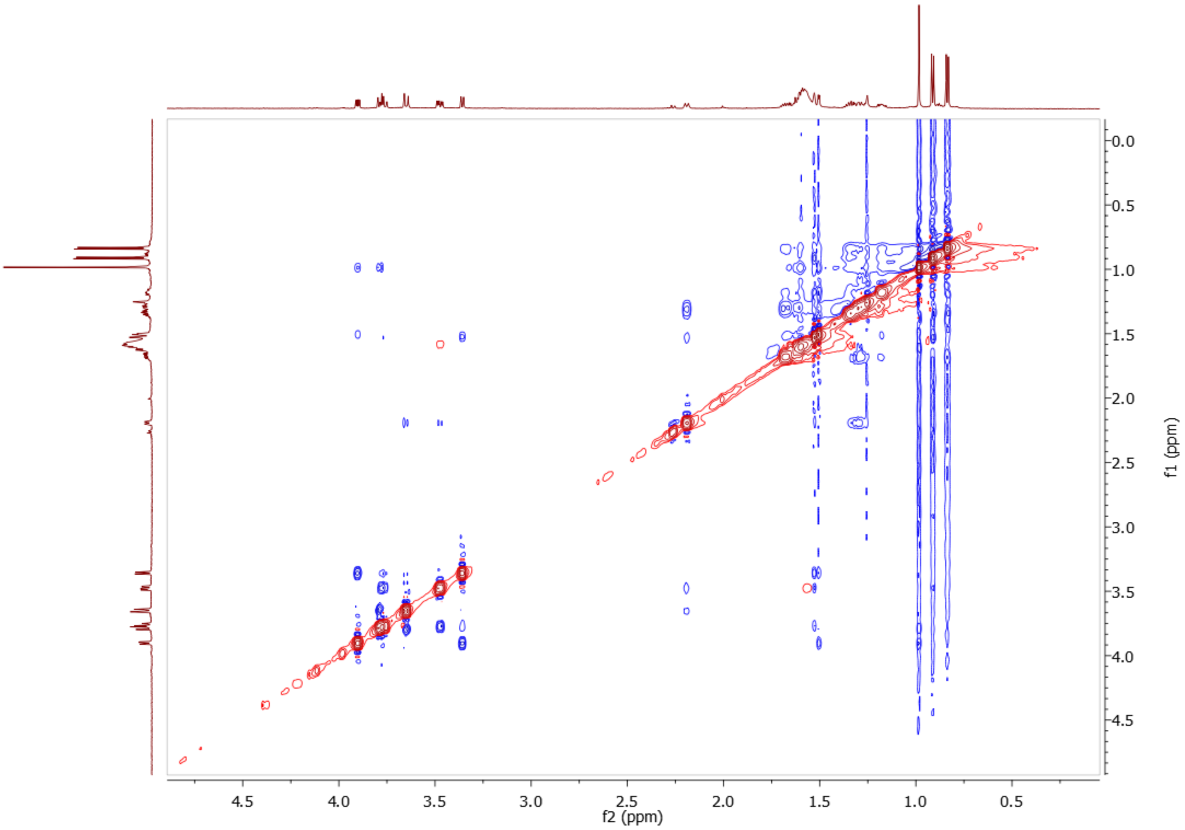

Figure S33: NOESY spectrum (600 MHz) of **4** in $CDCl_3$

Elemental Composition Report

Single Mass Analysis
Tolerance = 10.0 PPM / DBE: min = -1.5, max = 50.0
Element prediction: Off
Number of isotope peaks used for i-FIT = 3

Monoisotopic Mass, Even Electron Ions
31 formula(e) evaluated with 1 results within limits (up to 50 best isotopic matches for each mass)
Elements Used:
C: 0-50 H: 0-50 O: 0-10
BF14-1-103-May-2019BF14-1-1
BF14-1-1
BF14-1-1_4 10 (0.212)
1: TOF MS ES+
1.11e+003

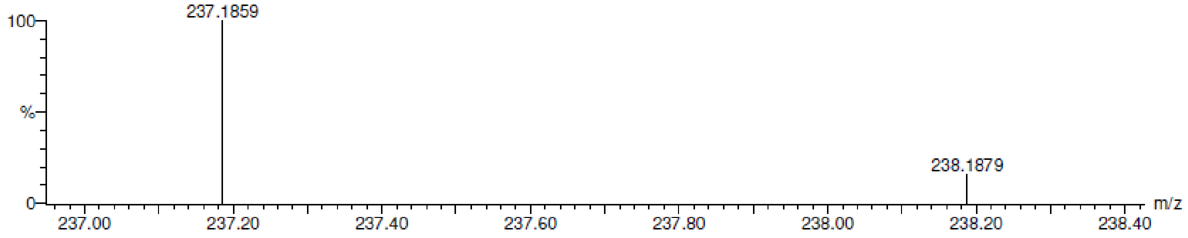

Minimum:				-1.5			
Maximum:		5.0	10.0	50.0			
Mass	Calc. Mass	mDa	PPM	DBE	i-FIT	i-FIT (Norm)	Formula
237.1859	237.1855	0.4	1.7	3.5	16.7	0.0	C15 H25 O2

Figure S34: HRESIMS spectrum of **4**

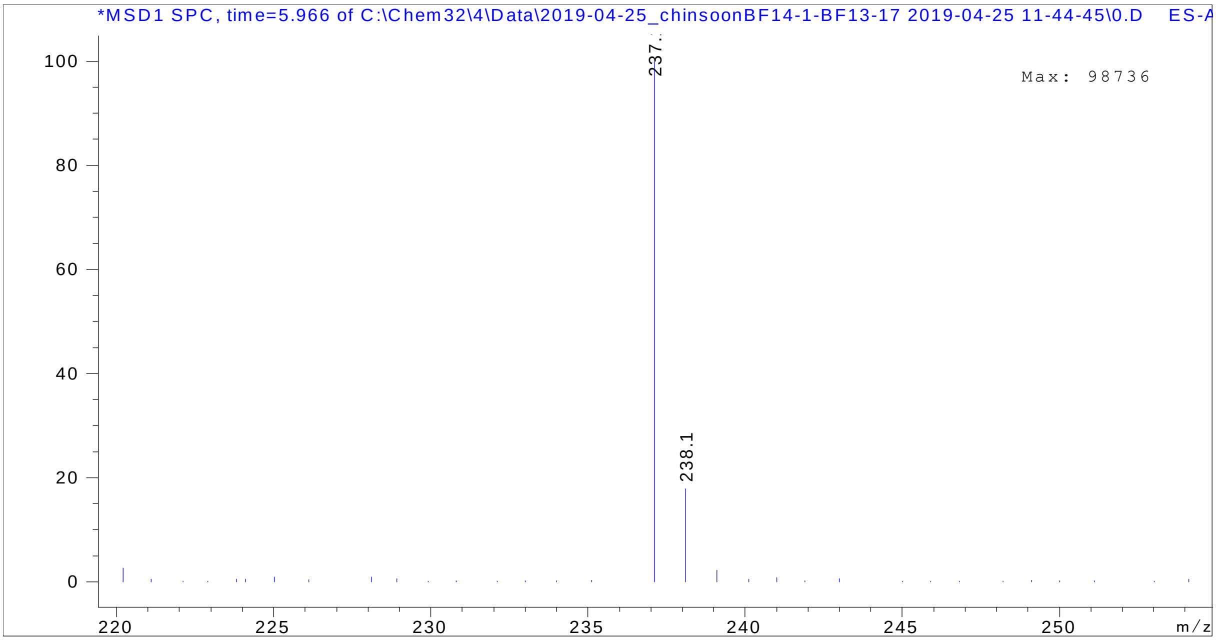

Figure S35: ESIMS spectrum of **4**

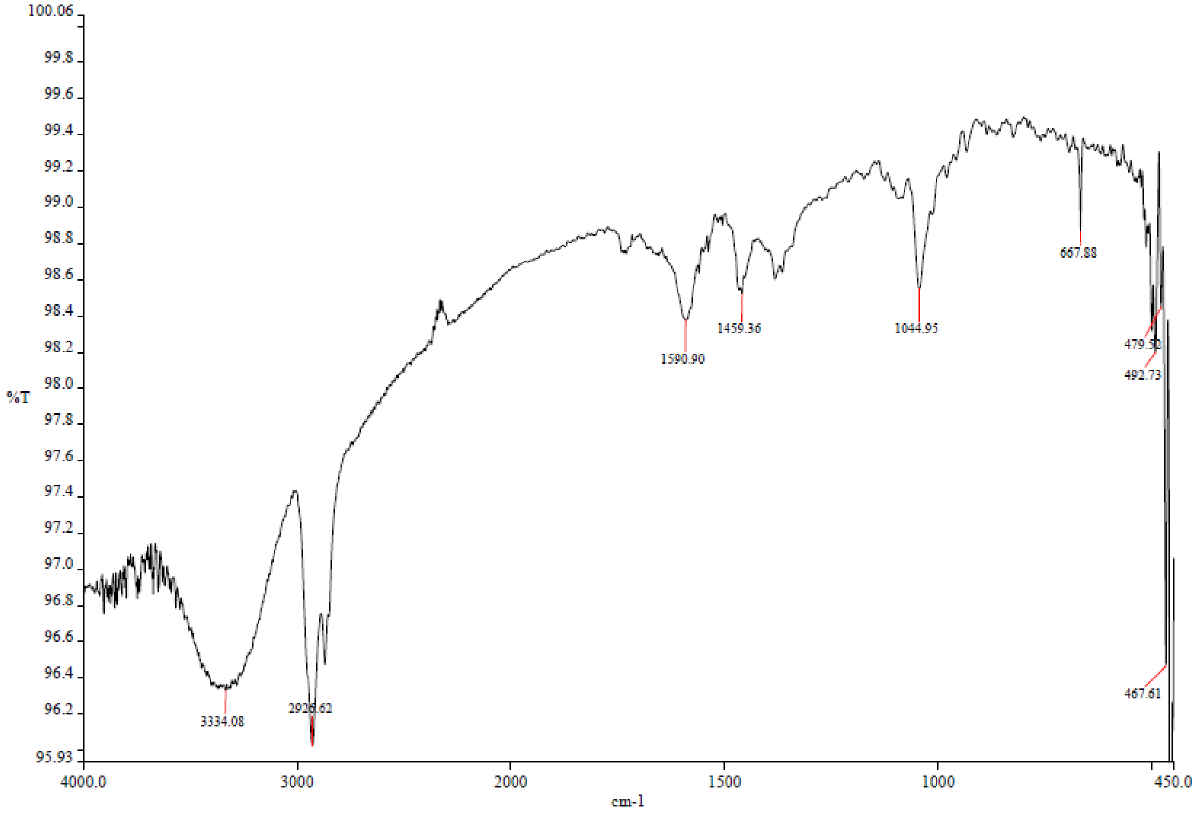

Figure S36: IR spectrum of **4**

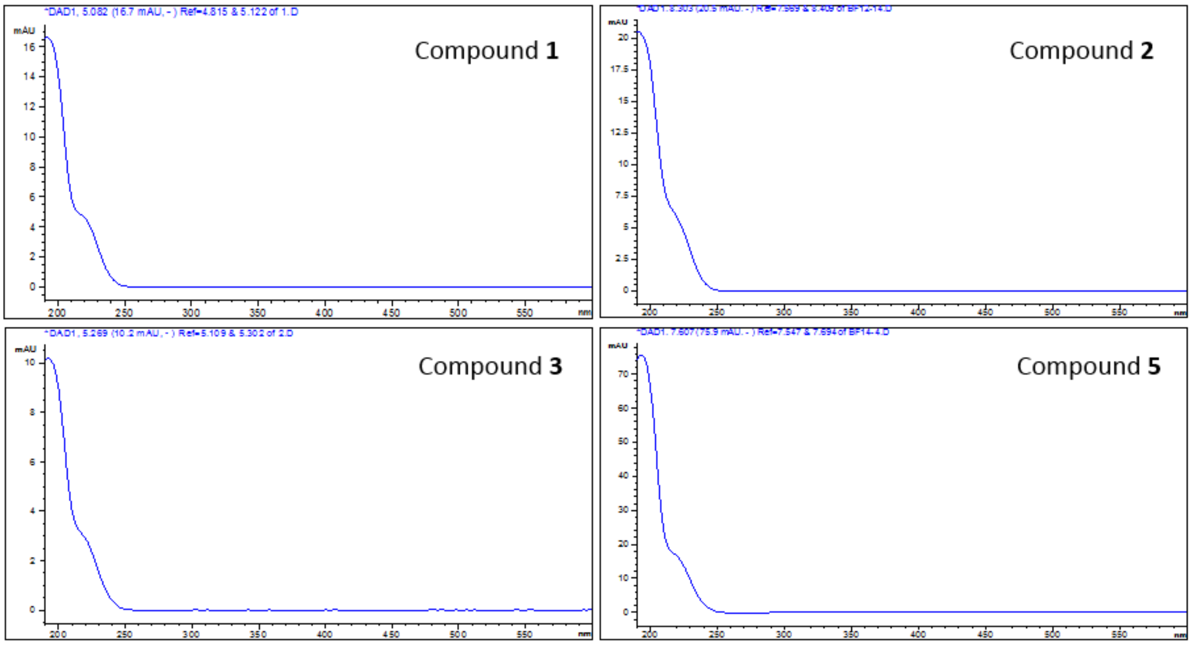

Figure S37: UV-vis spectrum of **1–3** and **5**

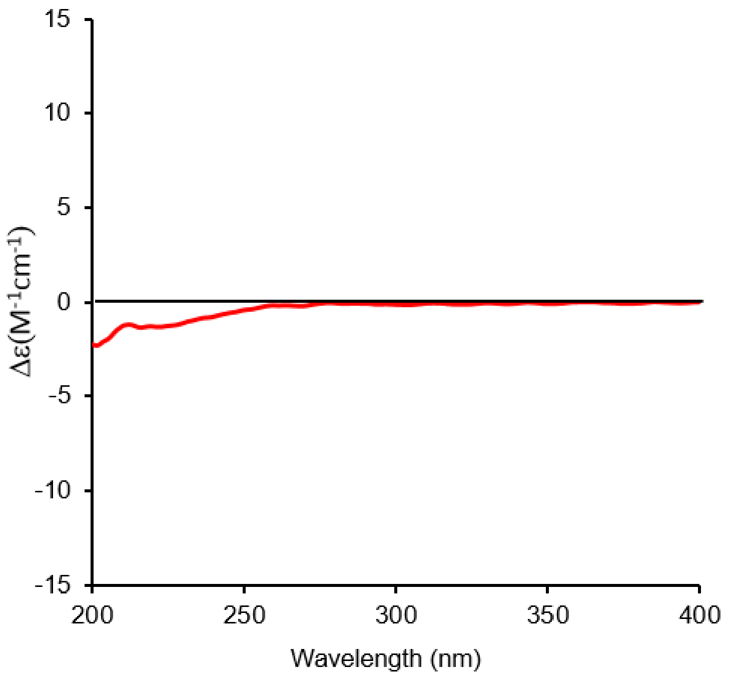

Figure S38: ECD spectrum of **4**

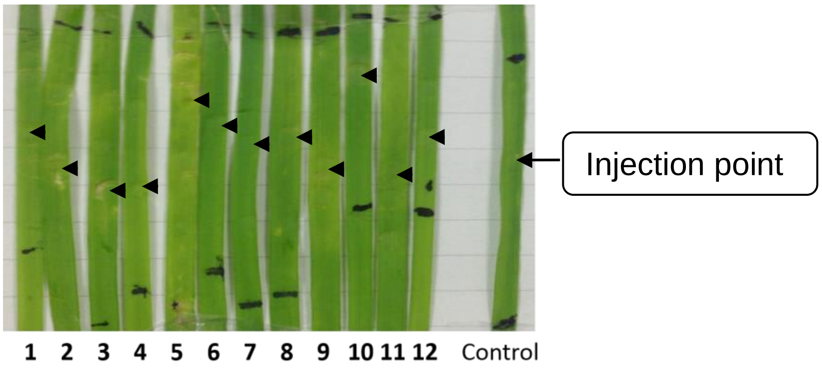

Figure S39: Phytotoxic activity of **1–12** against wheat leaves at 200 ppm

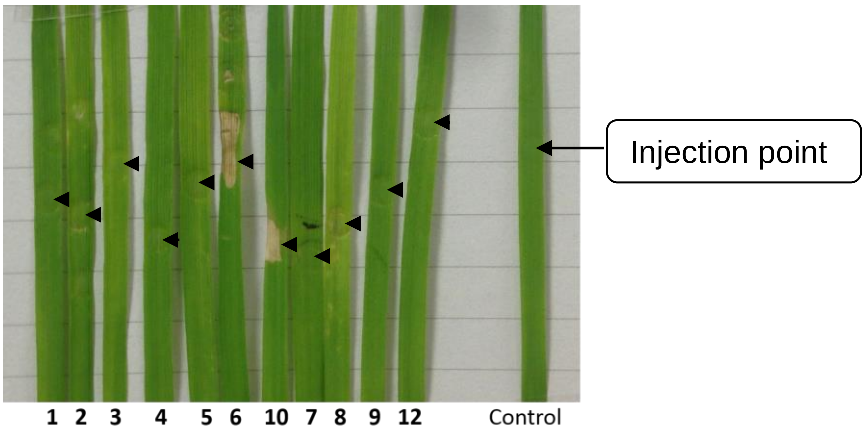

Figure S40: Phytotoxic activity of **1–10** and **12** against wheat leaves at 500 ppm

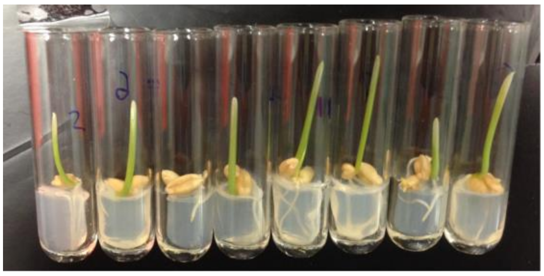

1 6 7 8 9 10 12 Control

Figure S41: Phytotoxic activity of **1**, **6–10** and **12** against wheat germination at 100 ppm on day 5

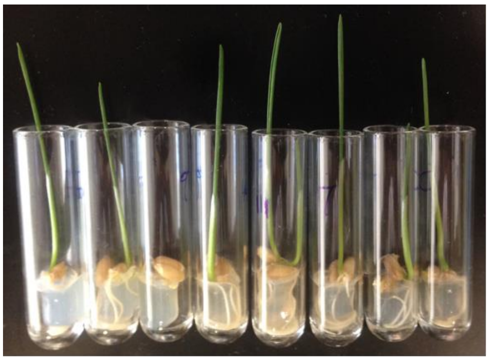

1 6 7 8 9 10 12 Control

Figure S42: Phytotoxic activity of **1**, **6–10** and **12** against wheat germination at 100 ppm on day 7
